# Chicken bile powder protects against α-naphthylisothiocyanate-induced cholestatic liver injury in mice

**DOI:** 10.18632/oncotarget.21385

**Published:** 2017-09-27

**Authors:** Yi-Fei Li, Jia-Sheng Wu, Yuan-Yuan Li, Yan Dai, Min Zheng, Jia-Kai Zeng, Guo-Feng Wang, Tian-Ming Wang, Wen-Kai Li, Xue-Yan Zhang, Ming Gu, Cheng Huang, Li Yang, Zheng-Tao Wang, Yue-Ming Ma

**Affiliations:** ^1^ Department of Pharmacology, School of Pharmacy, Shanghai University of Traditional Chinese Medicine, Shanghai 201203, China; ^2^ School of Pharmacy, Shanghai University of Traditional Chinese Medicine, Shanghai 201203, China; ^3^ Research Centre for Traditional Chinese Medicine of Complexity Systems, Shanghai University of Traditional Chinese Medicine, Shanghai, China; ^4^ Shanghai Key Laboratory of Complex Prescription and MOE Key Laboratory for Standardization of Chinese Medicines, Institute of Chinese Materia Medica, Shanghai University of Traditional Chinese Medicine, Shanghai 201203, China; ^5^ Shanghai Key Laboratory of Compound Chinese Medicines, Shanghai University of Traditional Chinese Medicine, Shanghai 201203, China

**Keywords:** intrahepatic cholestasis, chicken bile powder, FXR, bile acid, metabolomics

## Abstract

This study explored the effects of chicken bile powder (CBP), a 2000-year-old Chinese medicine, on α-naphthyl isothiocyanate (ANIT)-induced intrahepatic cholestasis in mice. CBP treatment for 14 days significantly ameliorated ANIT-induced changes in serum alanine aminotransferase, aspartate aminotransferase, bile acids, bilirubin, γ-glutamyl transpeptidase, alkaline phosphatase, and liver tissue morphology. Serum metabolomics showed changes in 24 metabolites in ANIT-exposed mice; 16 of these metabolites were reversed by CBP treatment via two main pathways (bile acid biosynthesis and arachidonic acid metabolism). Additionally, CBP administration markedly increased fecal and biliary bile acid excretion, and reduced total and hydrophobic bile acid levels in the livers of cholestatic mice. Moreover, CBP increased liver expression of bile acid efflux transporters and metabolic enzymes. It also attenuated ANIT-induced increases in hepatic nuclear factor-κB-mediated inflammatory signaling, and increased liver expression of the nuclear farnesoid X receptor (FXR) in cholestatic mice. CBP also activated FXR *in vitro* in HEK293T cells expressing mouse Na^+^-taurocholate cotransporting polypeptide. It did not ameliorate the ANIT-induced liver injuries in FXR-knockout mice. These results suggested that CBP provided protection from cholestatic liver injury by restoring bile acid homeostasis and reducing inflammation in a FXR-dependent manner.

## INTRODUCTION

Intrahepatic cholestasis is a clinical syndrome associated with the systemic and intrahepatic accumulation of toxic bile acids (BAs), which ultimately cause hepatobiliary injury [1]. Several liver diseases can develop due to intrahepatic cholestasis, including biliary atresia, primary sclerosing cholangitis, and primary biliary cirrhosis [2]. The natural BA ursodeoxycholic acid (UDCA) is the first and currently most established medical treatment for intrahepatic cholestasis, and this has been used to treat the early stages of primary biliary cirrhosis [3]. However, some patients do not respond to UDCA [4]. Therefore, the development of novel therapeutic approaches to cholestatic liver disorders is required.

The enterohepatic circulation of BAs is mediated by a number of metabolic enzymes and transporters [5, 6]. Drug intervention [7–10], pregnancy [11], and gene mutation [12, 13] can alter the expression or function of efflux transporters and metabolic enzymes. This can result in the hepatic retention of toxic BAs, which can damage the liver by inducing mitochondrial dysfunction and hepatocyte apoptosis or necrosis [14, 15]. Recent studies have shown that nuclear receptors can regulate these BA transporters and metabolic enzymes, and thus play an important role in maintaining BA homeostasis [16, 17]. The farnesoid X receptor (FXR) is one of the important nuclear receptors in this context. Activation of FXR provides multiple benefits that can reduce cholestatic liver injury, including the induction of efflux transporters and modulation of metabolic enzyme expression [18, 19]. FXR activation can also decrease hepatic inflammation mediated by the nuclear factor (NF)-κΒ signaling pathway [20, 21]. Therefore, activation of FXR represents a therapeutic target for intrahepatic cholestasis.

Traditional medicines and natural products have been widely used to treat cholestasis for thousands of years [22–24]. Dried black bear’s bile was recommended in China for the treatment of jaundice during the Tang dynasty (618-907 A.D.), as documented in the Tang Materia Medica, the first state pharmacopoeia [25]. However, the application of bear’s bile is greatly restricted by its availability and by ethical considerations. Chicken bile (CB), another ancient traditional Chinese medicine, was first recorded in the classic book “Ming Yi Bie Lu”, and has been used to treat respiratory and ophthalmological conditions since the Han Dynasty [26]. However, it is not known whether CB has protective effects against cholestatic liver injury. CB consists of some BAs, notably taurochenodeoxycholic acid (TCDCA; around 80%) and taurocholic acid (TCA; around 17%) [27]. TCDCA can be converted into chenodeoxycholic acid (CDCA) by intestinal bacteria [28]. It has been confirmed that CDCA is the main FXR ligand [16]. Therefore, we wished to investigate whether CB powder (CBP) protected against the liver damage induced by cholestasis, and whether this involved FXR activation. The purpose of this study was to address these questions in mice with α-naphthyl isothiocyanate (ANIT)-induced cholestasis. Our results might provide novel insights into the potential of novel naturally occurring compounds to treat intrahepatic cholestasis.

## RESULTS

### CBP ameliorates ANIT-induced cholestatic liver injury

**Figure 1:**
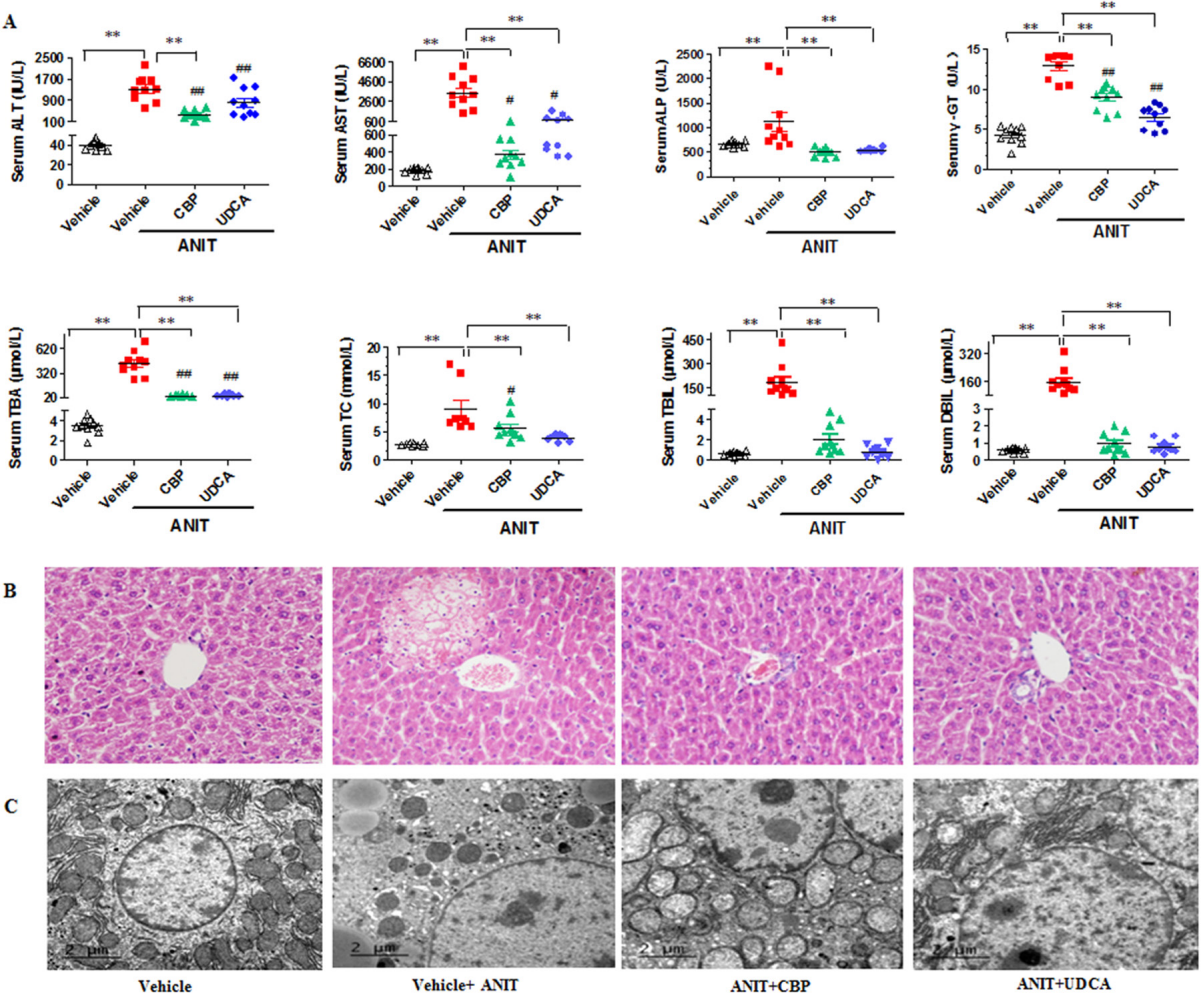
CBP ameliorates ANIT-induced intrahepatic cholestasis in mice **(A)** Serum biochemical parameters. **(B)** Liver morphology was observed by light microscopy (original magnification × 200), and **(C)** microstructure was observed by electron microscopy (scale bar 2 μm, original magnification × 6000). Data are expressed as the mean ± SD, n = 10 for serum biochemical parameters and light microscopy, and n = 3 for electron microscopy; ^**^p < 0.01 for the comparison with the vehicle + ANIT group; ^#^p < 0.05, ^##^p < 0.01 for the comparison with the vehicle group.

As shown in Figure 1A, serum alanine aminotransferase (ALT) and aspartate aminotransferase (AST), which provide biochemical indicators of hepatic cell damage, were increased in ANIT-induced mice. CBP treatment dose-dependently reduced these increases (Figure 1A and Supplementary Figure 1). Likewise, CBP administration reversed ANIT-induced increases in serum alkaline phosphatase (ALP), γ-glutamyl transpeptidase (γ-GT), total bilirubin (TBIL), direct bilirubin (DBIL), and total BAs (TBA); these are all biomarkers of cholestasis (Figure 1A and Supplementary Figure 1). In addition, light microscopy showed that the liver tissue of the vehicle-treated group exhibited a normal structure (Figure 1B). However, liver tissue specimens from the ANIT group showed acute inflammatory cell infiltration, edema, and hepatic necrosis (Figure 1B). ANIT mice treated with CBP exhibited a mild degree of hepatocyte hydropic degeneration, with less inflammatory cell infiltration (Figure 1B). Abnormal dilatation of the endoplasmic reticulum was found in the ANIT-treated group using electron microscopic analysis, and this was significantly ameliorated by CBP (Figure 1C). As shown in Supplementary Figure 2, CBP treatment had no effect on liver function or the endoplasmic reticulum in normal mice. Taken together, these results suggested that CBP alleviated the cholestatic liver injury induced by ANIT.

### Serum metabolomics study of CBP in cholestatic mice

**Figure 2:**
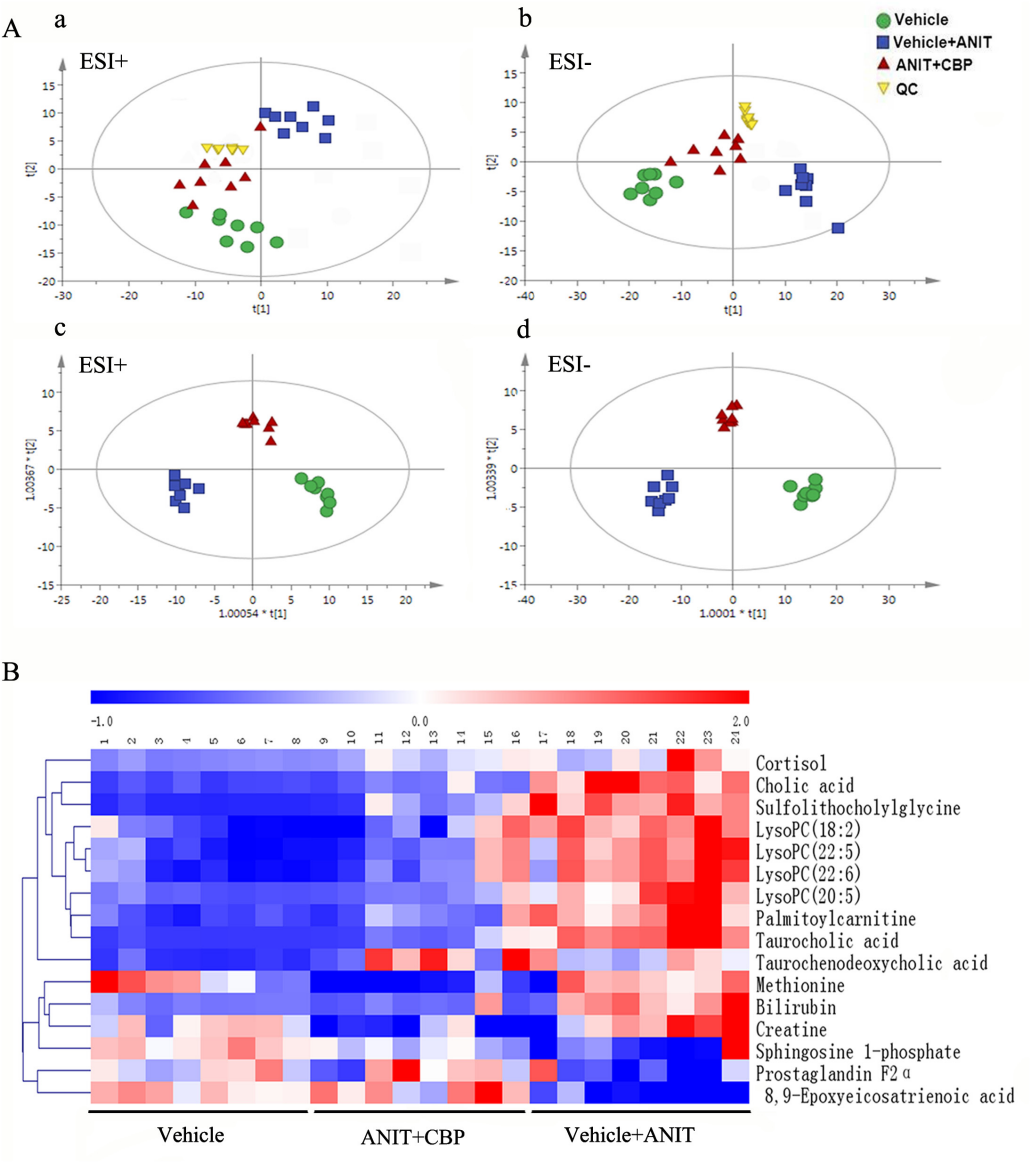
Metabolomics study of CBP in mice with intrahepatic cholestasis **(A-a)** PCA score plot in positive ion mode, R^2^X = 0.94, Q^2^ = 0.835; **(A-b)** PCA score plot in negative ion mode, R^2^X = 0.942, Q^2^ = 0.792. **(A-c)** OPLS-DA score plot in positive ion mode, R^2^X = 0.9, R^2^Y = 0.947, Q^2^ = 0.851; **(A-d)** OPLS-DA score plot in negative ion mode. R^2^X = 0.852, R^2^Y = 0.967, Q^2^ = 0.849. **(B)** Hierarchical clustering heat map of the metabolites with differential levels in the vehicle, ANIT+CBP, and vehicle+ANIT groups.

The quality control (QC) samples clustered tightly in positive and negative modes in the principal components analysis (PCA) plot (Figure 2Aa and 2Ab), illustrating the stability of the liquid chromatography/mass spectrometry method throughout the whole run (methodology validation is shown in Supplementary Table 1). Orthogonal partial least squares discriminant analysis (OPLS-DA) was employed to compare the vehicle- and ANIT-treated groups (Supplementary Figure 3A-3B), and the ANIT- and ANIT+CBP-treated groups (Supplementary Figure 3C-3D); this facilitated the identification of potential differential metabolites. After screening with “VIP > 1.00” and “p < 0.05”, 211 (ESI+) and 293 (ESI-) differential metabolites were identified between the vehicle- and ANIT-treated groups, while 239 (ESI+) and 347 (ESI-) differential metabolites were identified between the ANIT- and ANIT+CBP-treated groups. Figure 2Ac and 2Ad shows the OPLS-DA results, indicating an appreciable separation of the data relating to these three groups. The model statistics, R^2^X, R^2^Y, and Q^2^, indicated that the models were stable and reliable, with high predictive ability.

#### Identification of differential metabolites

The differential metabolites indicated by the comparison between each pair of groups are presented in Supplementary Tables 2 and 3. As compared with the normal control group, 24 serum metabolites showed significantly different levels in the ANIT group and 16 of these differences were reversed by CBP intervention (Table 1). Seven of the 16 differential metabolites were identified by authenticated standards, and the others were deduced on the basis of accurate molecular weights, tandem mass spectrometry fragments, and metabolomics databases. The metabolites that showed significant changes included amino acids, BAs, fatty acids, phospholipids, and inflammatory mediators. Figure 2B provides a heat map showing how the levels of these metabolites varied among these three study groups.

**Table 1:** Endogenous metabolites identified in the serum of mice included in this study

VIP	Time	Molecular ion	Compound	MZ	Formula	Metabolites	HMDB
	(min)		MW				
1.2371	0.87	[M+H]^+^	131.0688	132.0761	C_4_H_9_N_3_O_2_	Creatine^*^	00064
1.9915	0.95	[M+H]^+^	149.0503	150.0575	C_5_H_11_NO_2_S	Methionine^*^	00696
3.9315	3.77	[M+H]^+^	515.2887	516.2960	C_26_H_45_NO_7_S	Taurocholic acid^*^	00036
2.6512	3.91	[M-H]^−^	513.2745	512.2678	C_26_H_43_NO_7_S	Sulfolithocholylglycine	02639
2.3625	4.40	[M+H]^+^	362.2077	363.2149	C_21_H_30_O_5_	Cortisol	00063
1.5510	4.41	[M-H]^−^	362.2084	361.2015	C_21_H_30_O_5_	Cortisol	00063
1.9255	4.58	[M-H]^−^	408.2865	407.2794	C_24_H_40_O_5_	Cholic acid^*^	00619
3.1122	4.93	[M+H]^+^	584.2606	585.2679	C_33_H_36_N_4_O_6_	Bilirubin^*^	00054
3.7841	5.04	[M+H]^+^	499.2948	500.3046	C_26_H_45_NO_6_S	Taurochenodeoxycholic acid^*^	00951
1.2292	5.46	[M-H]^−^	354.2397	353.2335	C_20_H_34_O_5_	Prostaglandin F2α^*^	01139
1.4079	6.92	[M+H]^+^	379.2470	380.2542	C_18_H_38_NO_5_P	Sphingosine 1-phosphate	00277
1.0619	6.92	[M-H]^−^	379.2477	378.2406	C_18_H_38_NO_5_P	Sphingosine 1-phosphate	00277
2.3307	7.10	[M+H]^+^	541.3142	542.3215	C_28_H_48_NO_7_P	LysoPC(20:5)	10397
1.4450	7.54	[M+H]^+^	519.3282	520.3354	C_26_H_50_NO_7_P	LysoPC(18:2)	10386
1.5763	7.67	[M+H]^+^	567.3278	568.3351	C_30_H_50_NO_7_P	LysoPC(22:6)	10404
1.8473	7.97	[M+H]^+^	569.3453	570.3525	C_30_H_52_NO_7_P	LysoPC(22:5)	10403
1.5650	8.40	[M-H]^−^	320.2344	319.2276	C_20_H_32_O_3_	8,9-Epoxyeicosatrienoic acid	02232
1.5343	8.61	[M+H]^+^	399.3314	400.3387	C_23_H_45_NO_4_	L-Palmitoylcarnitine	00222

^*^ Identified by authenticated standard.

#### Analysis of the metabolic pathways affected by CBP

In order to explore the possible metabolic pathways that were affected by CBP in mice with cholestasis, the 16 endogenous metabolites that were affected by ANIT and reversed by CBP were imported into Metaboanalyst. The main metabolic pathways that these molecules were associated with included primary BA biosynthesis, arachidonic acid metabolism, sphingolipid metabolism, cysteine and methionine metabolism, taurine and hypotaurine metabolism, and steroid hormone biosynthesis (Supplementary Table 4). The top two pathways influenced by CBP were primary BA biosynthesis and arachidonic acid metabolism (Supplementary Table 4).

### CBP reverses disordered BA homeostasis in cholestatic mice

**Figure 3:**
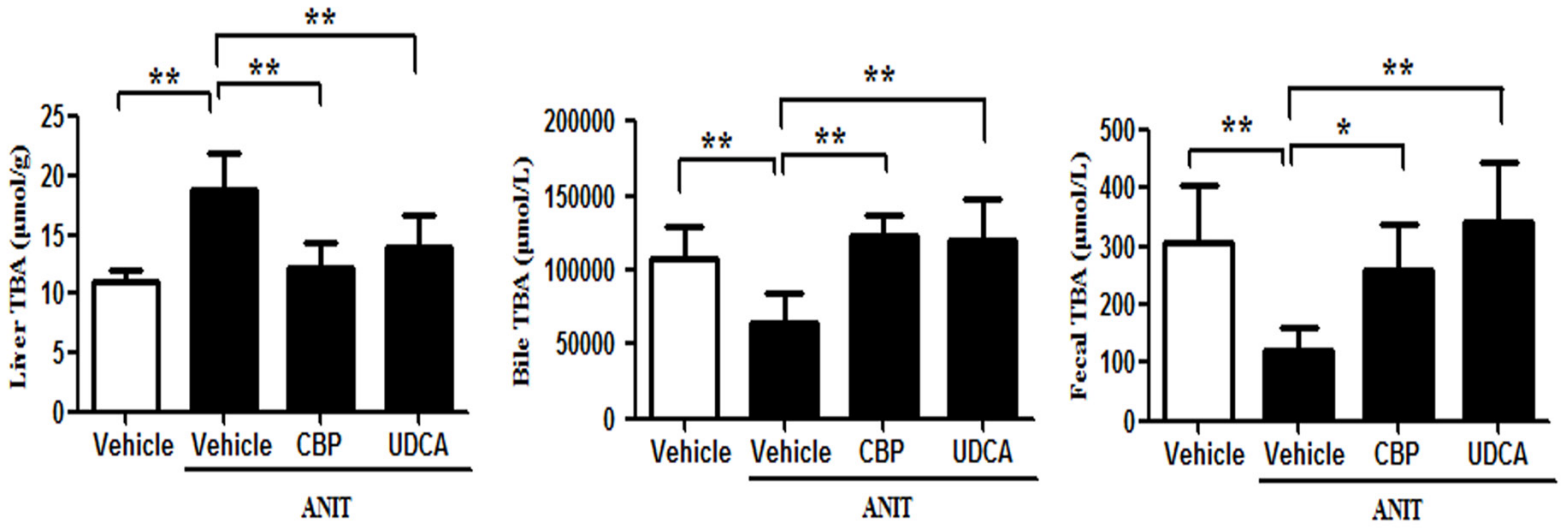
CBP decreases the liver, bile, and fecal total BA levels in mice with intrahepatic cholestasis Data are expressed as the mean ± SD, n = 10; ^*^p < 0.05, ^**^p < 0.01 for the comparison with the vehicle+ANIT group.

**Figure 4:**
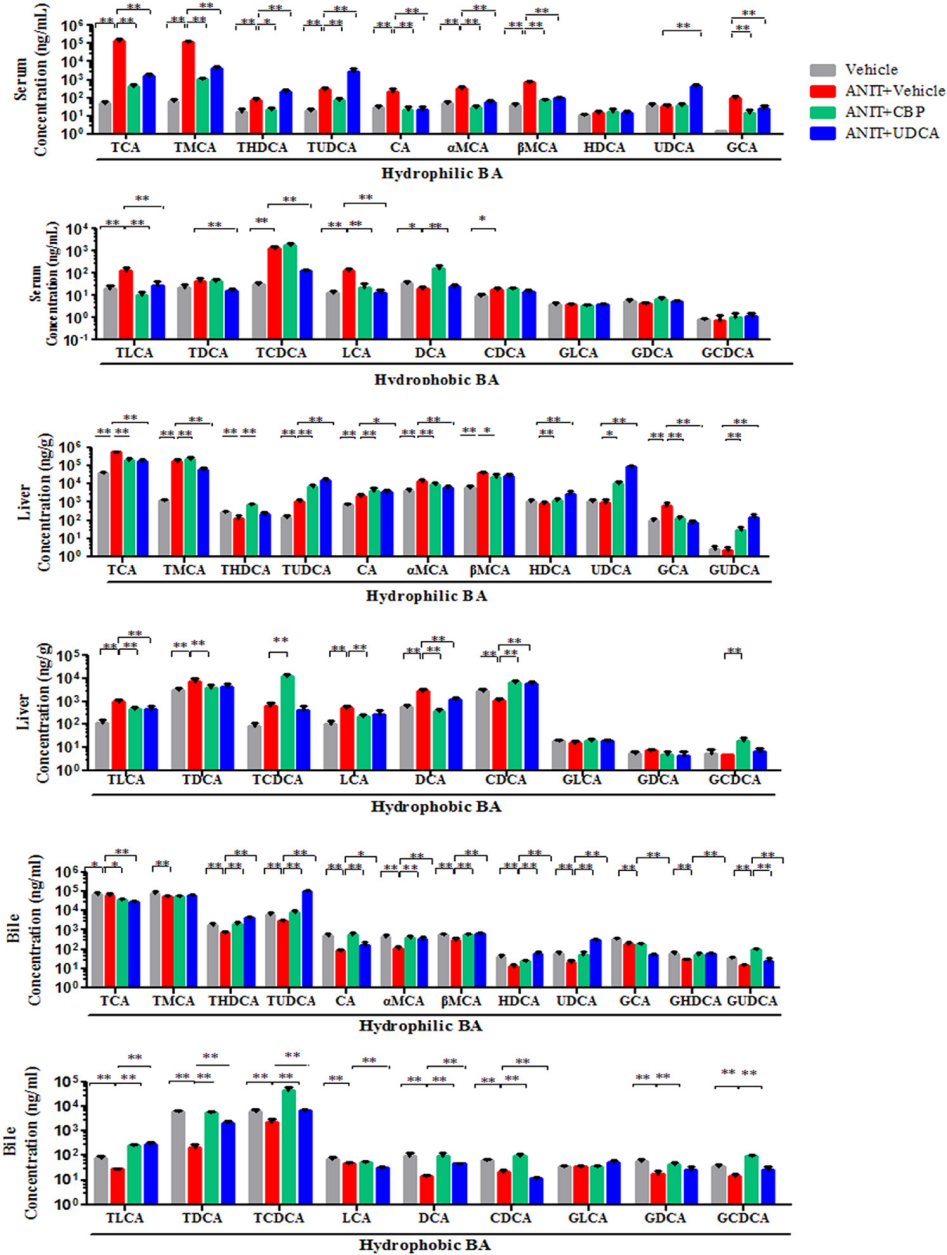
The influence of CBP on hydrophilic and hydrophobic BAs detected by UPLC-MS/MS in mice with intrahepatic cholestasis Data are expressed as the mean ± SD, n = 10; ^*^p < 0.05, ^**^p < 0.01 for the comparison with the vehicle+ANIT group.

As compared with the vehicle group, the total serum and liver BA levels were significantly increased in the ANIT-treated group. However, CBP treatment reduced the TBA level in serum (Figure 1A) and liver (Figure 3). Moreover, 18 individual BA levels in mouse serum, liver, and bile were shown in Figure 4. In cholestatic mice, CBP treatment decreased the hepatic levels of hydrophobic BAs, such as deoxycholic acid (DCA), lithocholic acid (LCA), taurodeoxycholic acid (TDCA), and taurolithocholic acid (TLCA), and increased the hydrophilic BAs, such as UDCA and tauroursodeoxycholic acid (TUDCA; Figure 4). Levels of TCDCA and CDCA were also increased in the livers of cholestatic mice. Additionally, CBP treatment significantly increased the total biliary and fecal BA concentrations (Figure 3). Moreover, CBP treatment significantly increased the liver levels of UDCA, CDCA, TCDCA, and TUDCA in normal mice (Supplementary Figure 5).

### CBP increases expression of FXR, BA transporters, and metabolic enzymes in cholestatic mice

**Figure 5:**
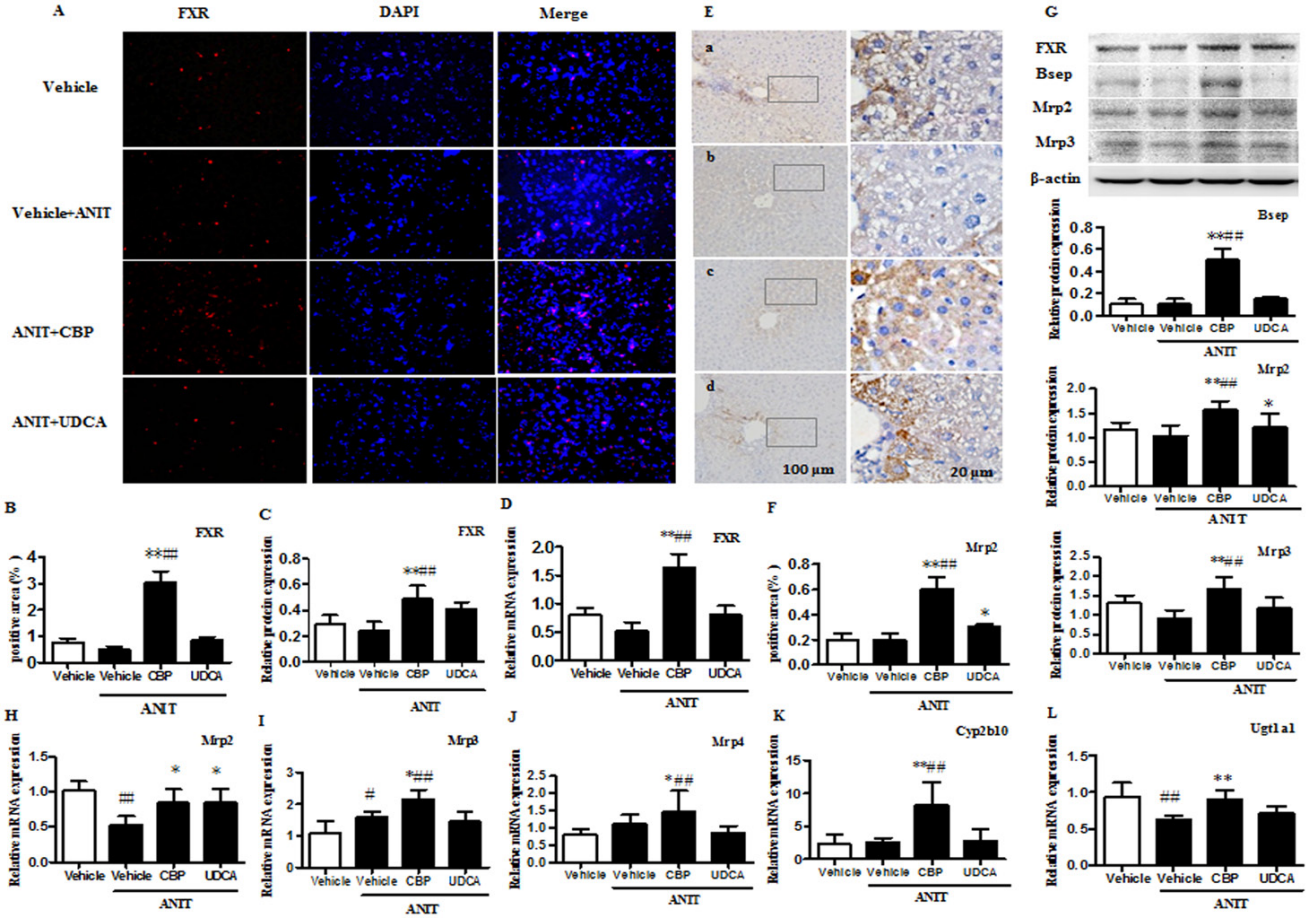
CBP increases expression of FXR, BA transporters, and metabolic enzymes in mice with intrahepatic cholestasis **(A)** Effect of CBP on FXR protein expression, detected by immunofluorescence. **(B-D)** Quantitative analysis of liver FXR expression, detected by immunofluorescence, western blot, and real-time PCR. **(E-F)** Effect of CBP on liver expression of Mrp2, detected by immunohistochemistry. **(G)** Effect of CBP on FXR, Bsep, Mrp2, and Mrp3 protein expression, detected by western blot. **(H-L)** Effect of CBP on Mrp2, Mrp3, Mrp4, Cyp2b10, and Ugt1a1 mRNA levels, detected by real-time PCR. Data are expressed as the mean ± SD, ^*^p < 0.05, ^**^p < 0.01 for the comparison with the vehicle+ANIT group; ^#^p < 0.05, ^##^p < 0.01 for the comparison with the vehicle group.

As shown in Figure 5A-5D and 5G, CBP treatment markedly increased the mRNA and protein expression of FXR in the livers of mice with ANIT-induced cholestasis. Treatment with CBP for 14 days had slightly increased the FXR protein expression in normal mice (Supplementary Figure 6). Additionally, CBP treatment also has no influence on the protein expression of FXR in mice that induced by ANIT at 24h (Supplementary Figure 7). The BA efflux transporter, Bsep, which is the main target gene of FXR, also showed significantly elevated protein expression in the livers of cholestatic mice treated with CBP (Figure 5G). Moreover, as shown in Figure 5E-5G, CBP administration significantly increased the liver protein expression of multidrug resistance-associated protein (Mrp) 2, and prevented the ANIT-induced decrease in Mrp2 mRNA expression (Figure 5H). Notably, CBP treatment also significantly increased hepatic expression of Mrp3 protein (Figure 5G), and the expression of Mrp3 and Mrp4 mRNAs (Figure 5I-5J). In addition to affecting BA transporters, CBP treatment markedly increased liver expression of mRNAs encoding a BA hydroxylation enzyme (cytochrome P450 [Cyp] 2b10) and conjugation enzyme (UDP-glucuronosyltransferase 1A1 [Ugt1a1]) in cholestatic mice (Figure 5K-5L).

### CBP inhibits expression of hepatic inflammatory factors in cholestatic mice

**Figure 6:**
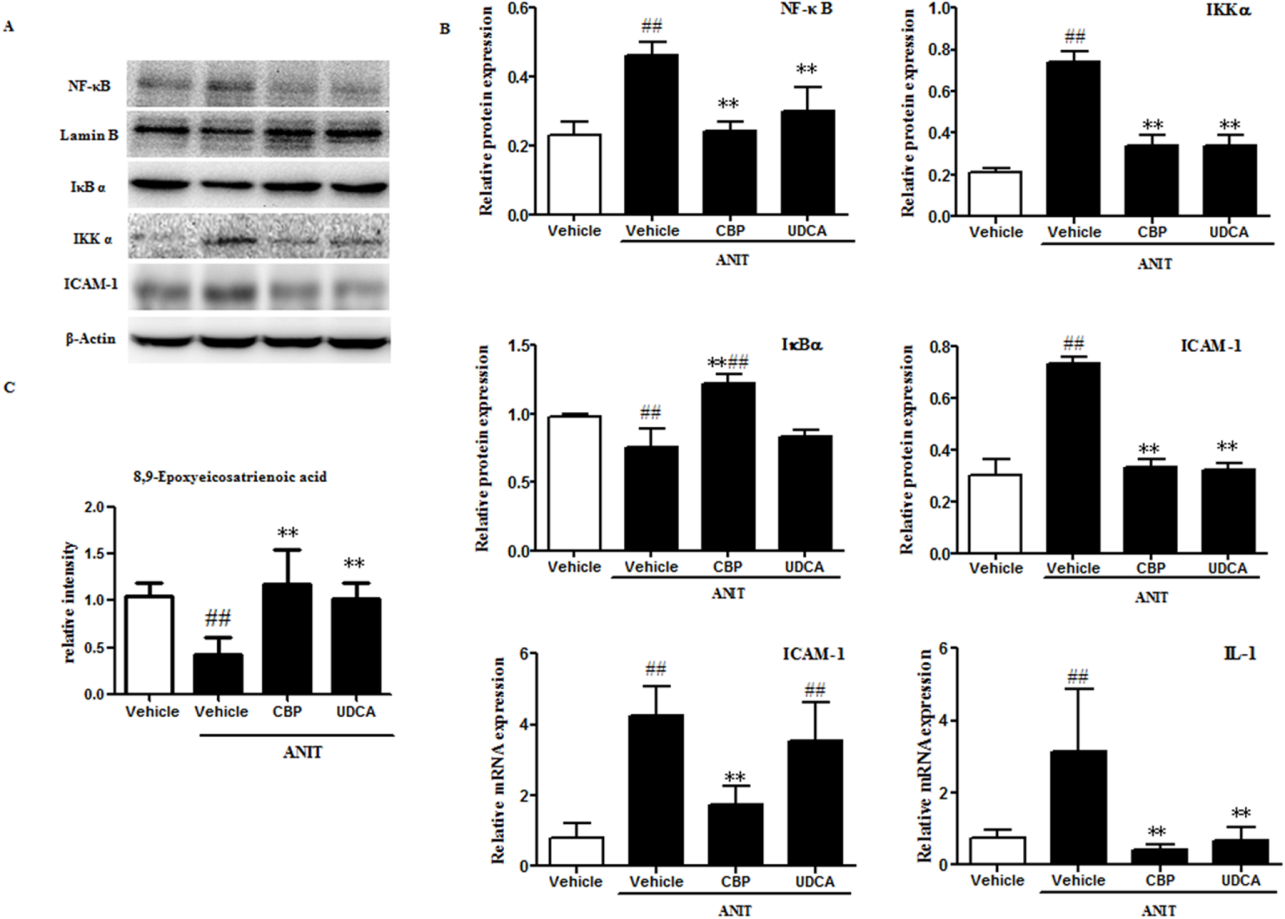
CBP inhibits liver inflammation in mice with intrahepatic cholestasis **(A)** The effects of CBP on protein levels of NF-κB, IκBα, IKKα, and ICAM-1, detected by western blot. **(B)** Quantitative analysis of the effects of CBP on NF-κB, IκBα, IKKα, and ICAM-1 proteins, and IL-1 and ICAM-1 mRNA expression. **(C)** Quantitative analysis of 8, 9-epoxyeicosatrienoic acid by UPLC-LTQ-orbitrap. Data are expressed as the mean ± SD, ^*^p < 0.05, ^**^p < 0.01 for the comparison with the vehicle+ANIT group; ^#^p < 0.05, ^##^p < 0.01 for the comparison with the vehicle group.

Metabolomics analysis showed that CBP treatment significantly increased the serum level of 8, 9-epoxyeicosatrienoic acid (Figure 6), as compared with the ANIT-induced cholestatic group. Additionally, as shown in Figure 6, the hepatic protein levels of inhibitor of kappa B kinase (IKK) α and NF-κB were significantly higher in the ANIT-induced cholestatic group than in the vehicle group, while inhibitor of kappa B α (IκBα) protein expression was decreased (Figure 6). Moreover, the liver levels of inflammatory factors such as interleukin-1 (IL-1) and intercellular adhesion molecule-1 (ICAM-1) were markedly increased in the ANIT-induced cholestatic group. However, treatment with CBP markedly decreased the expression of NF-κB and IKKα proteins, and increased the IκBα protein level. Notably, treatment with CBP significantly reduced the liver expression of IL-1 and ICAM-1 in cholestatic mice (Figure 6).

### CBP induces FXR activation in Na^+^-taurocholate cotransporting polypeptide (Ntcp)-transfected human embryonic kidney (HEK293T) cells

**Figure 7:**
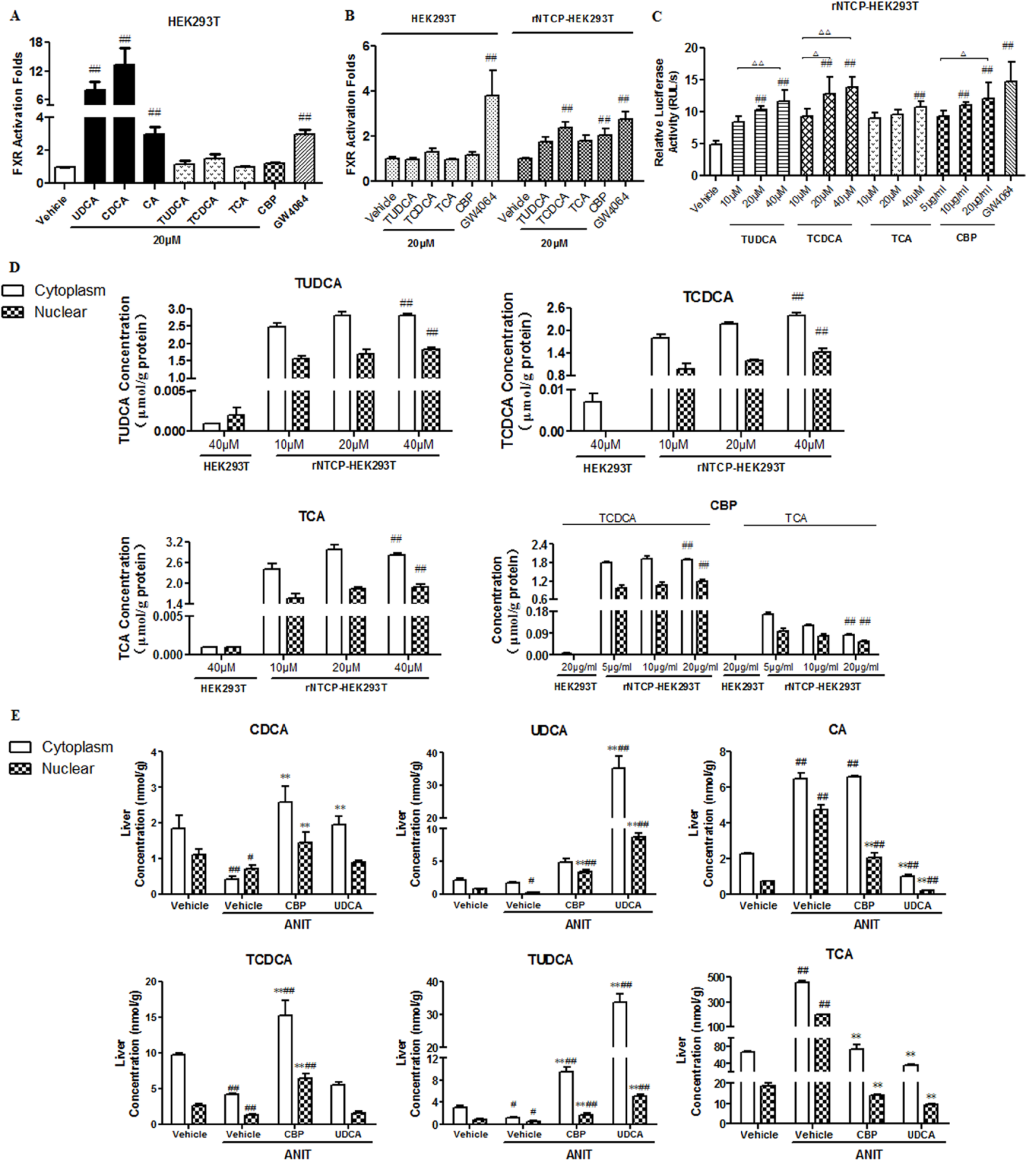
CBP promotes FXR activation in rNtcp-HEK293T cells and alters BA concentrations in rNtcp-HEK293T cells and mouse liver The effects of the indicated treatments on FXR activation in **(A)** HEK293T cells and **(B, C)** rNtcp-HEK293T cells. Data are expressed as the mean ± SD, n = 5; ^#^p < 0.05, ^##^p < 0.01 for the comparison with the vehicle group. **(D)** The cytoplasmic and nuclear BA concentrations in HEK293T and rNtcp-HEK293T cells exposed to the indicated treatments. Data are expressed as the mean ± SD, n = 3; ^#^p < 0.05, ^##^p < 0.01 for the comparison with the HEK293T cells (20 μg/ml treatments). **(E)** Cytoplasmic and nuclear BA concentrations in the livers of mice with intrahepatic cholestasis following treatment with CBP for two weeks. Data are expressed as the mean ± SD, n = 3; ^*^p < 0.05, ^**^p < 0.01 for the comparison with the vehicle+ANIT group; ^#^p < 0.05, ^##^p < 0.01 for the comparison with the vehicle group.

As shown in Figure 7, we performed dual luciferase assays to assess which components in CBP acted directly on the FXR. HEK293T cells were transfected with FXR, retinoid X receptor, and pRL-SV40 expression plasmids, in the presence or absence of a recombinant Ntcp (rNtcp) expression vector. Exposure to CDCA, UDCA, or cholic acid (CA) at 20 μM significantly induced FXR activity by 13-, 8-, and 3-fold in HEK293T cells, respectively (Figure 7A). However, CBP, TCDCA, TUDCA, and TCA did not produce this effect. Additionally, TCDCA and CBP both induced greater than 2-fold activation of FXR in rNtcp-HEK293T cells (Figure 7B). Moreover, TUDCA, TCDCA, and CBP concentration-dependently increased FXR activity in rNtcp-HEK293T cells (Figure 7C). To verify the source of this FXR activation effect, we performed separate quantitative analyses of BAs in the cytoplasm and nucleus. TUDCA, TCDCD, and TCA were detected in the cytoplasm and nucleus, whereas it was hard to detect UDCA, CDCA, and CA in rNtcp-HEK293T cells treated with TUDCA, TCDCD, or TCA, respectively (Figure 7D and Supplementary Table 6). Moreover, in rNtcp-HEK293T cells treated with CBP, TCDCA and TCA were detectable in the cytoplasm and nucleus, while CDCD and CA were not detected (Figure 7D and Supplementary Table 6).

### Effects of CBP on cytoplasmic and nuclear concentrations of BAs in liver tissues of cholestatic mice

This analysis indicated that UDCA, CDCA, CA, TUDCA, TCDCA, and TCA were all detected in the cytoplasm and nucleus of liver tissues from cholestatic mice treated with CBP (Figure 7E). The nuclear concentrations of CDCA, UDCA, TCDCA, and TUDCA were significantly higher in cholestatic mice treated with CBP, as compared with those treated with ANIT alone.

### Effects of CBP ingredients on ANIT-induced intrahepatic cholestasis in mice

As shown in Supplementary Figure 9, as compared with mice treated with ANIT alone, pretreatment with TCDCA significantly decreased the ANIT-induced increases in ALT, AST, γ-GT, TBA, TBIL, and DBIL in cholestatic mice. TCA could only decrease the increased levels of AST, TC, TBA, TBIL, and DBIL induced by ANIT, and had no influence on the ALT and γ-GT levels (Supplementary Figure 9).

### Effects of CBP on ANIT-induced intrahepatic cholestasis in FXR-knockout (FXRKO) mice

**Figure 8:**
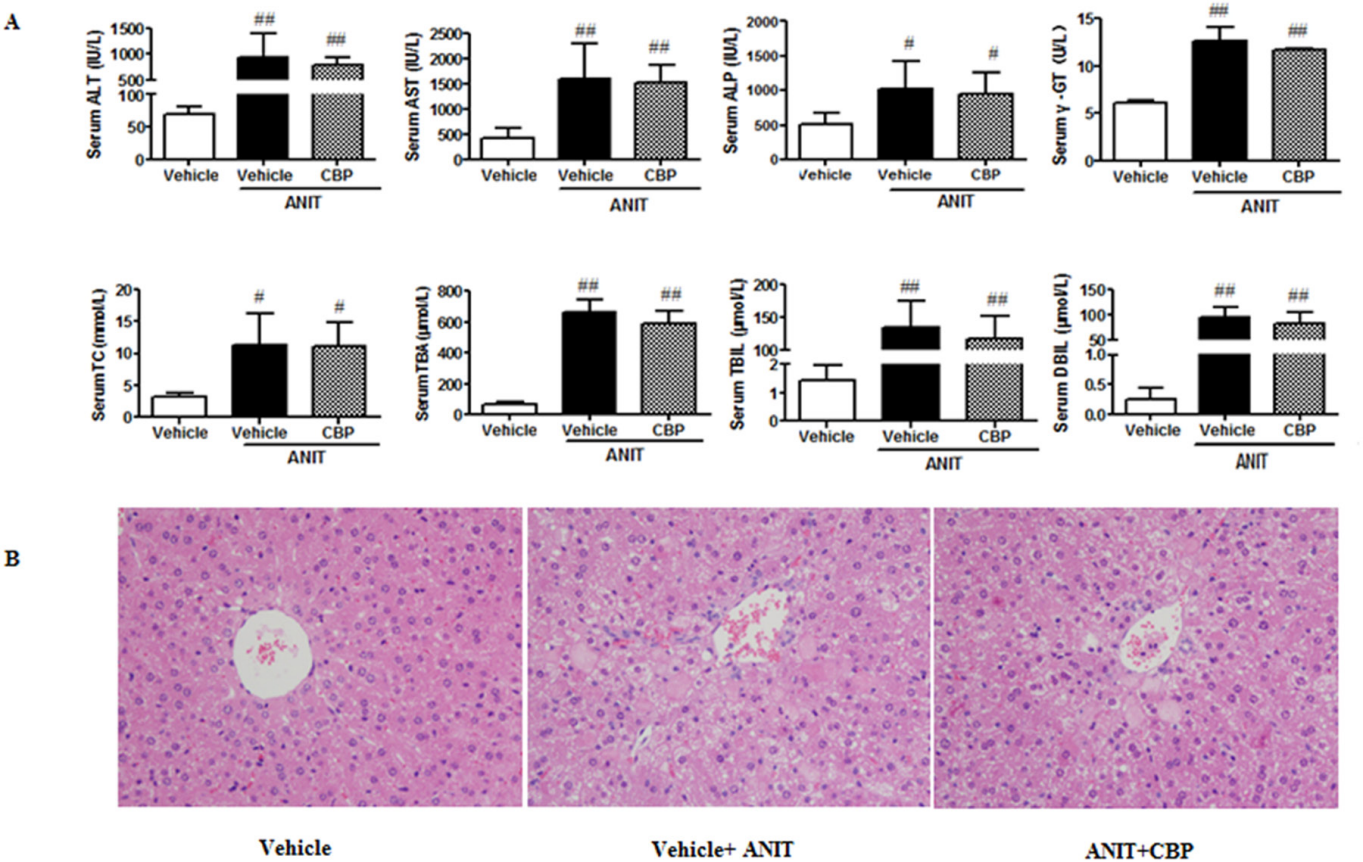
Effects of CBP on ANIT-induced intrahepatic cholestasis in FXRKO mice **(A)** Serum biochemical parameters. **(B)** Liver morphology was observed by light microscopy (original magnification × 200). Data are expressed as the mean ± SD, n = 4; ^#^p < 0.05, ^##^p < 0.01 for the comparison with the vehicle group.

As compared with the control group, the levels of ALT, AST, ALP, γ-GT, TC, TBA, TBIL, and DBIL were significantly increased in ANIT-induced cholestatic FXRKO mice. Treatment with CBP had no inhibitory effects on the ANIT-induced increases in these biochemical markers in cholestatic FXRKO mice (Figure 8A). In addition, hematoxylin-eosin staining identified acute inflammatory cell infiltration, edema, and hepatic necrosis in the livers of ANIT-induced cholestatic FXRKO mice, as compared with the control tissue, and CBP treatment did not ameliorate these changes (Figure 8B). These observations implied that CBP could not ameliorate ANIT-induced intrahepatic cholestasis in FXRKO mice.

## DISCUSSION

The use of bear’s bile is limited by availability and ethical concerns and it is therefore important to explore whether other natural products could be used to treat liver disorders. However, the effects of CBP on intrahepatic cholestatic liver disease had not been characterized prior to the present study. In recent years, mouse models have been used to evaluate the efficacy of traditional medicines [29–32]. In the current study, a mouse model of ANIT-induced intrahepatic cholestasis was used to investigate the effects of CBP on cholestatic liver injury. The present results found that CBP administration ameliorated ANIT-induced damage to liver histology and function, indicating that it has the potential to protect against cholestatic liver injury.

Metabolomic studies analyze the holistic and dynamic nature of biological systems, which is consistent with the core theory of holism and syndrome differentiation in traditional Chinese medicine [33, 34]. Therefore, metabolomics can help to clarify the metabolic pathways and mechanisms of action of traditional Chinese medicines [35–37]. In the present study, serum metabolomics found that BA biosynthesis and arachidonic acid metabolism were the two most important metabolic pathways that were influenced by CBP. This result suggested that the potential mechanism of action of CBP against intrahepatic cholestasis may associate with its influence on BA homeostasis and inflammation.

Overall, treatment with CBP ameliorated the high serum and liver levels of TBA found in the ANIT-treated mice and increased their biliary and fecal BA excretion. In addition, CBP administration decreased hepatocyte concentrations of toxic hydrophobic BAs such as DCA and LCA, which may aggravate hepatic apoptosis and necrosis [14, 15], and increased the levels of hydrophilic BAs such as UDCA and TUDCA, thus beneficially improving the hepatic BA composition. BAs are synthesized from cholesterol in the liver via reactions that are mainly catalyzed by Cyp7a1 and Cyp8b1. The current study identified reduced liver expression of Cyp7a1 and Cyp8b1 (Supplementary Figure 8), and increased liver cholesterol levels in ANIT-induced cholestatic mice, which may reflect intrahepatic BA retention. However, CBP treatment decreased the intrahepatic retention of BAs and reversed the effects of ANIT on liver Cyp7a1 and Cyp8b1 expression. Moreover, in the current study, CBP treatment induced the liver expression of Cyp2b10, Ugt1a1, and BA efflux transporters (Mrp2, Bsep, Mrp3, and Mrp4). These results suggested that CBP could increase the expression of specific BA metabolizing enzymes and efflux transporters, thereby promoting the detoxification and excretion of hepatotoxic BAs, thus reversing the disordered homeostasis of BAs.

Accumulation of toxic BAs has been reported to induce the production of inflammatory cytokines [38]. The present study observed decreased levels of epoxyeicosatrienoic acid in the ANIT-induced mouse liver. This metabolite of arachidonic acid has been reported to exert anti-inflammatory effects via a NF-κB-mediated mechanism [39–41]. In the current study, CBP treatment enhanced the serum level of 8, 9-epoxyeicosatrienoic acid in cholestatic mice, indicating that it may produce anti-inflammatory effects. Additional morphological analyses observed inflammatory cell infiltration in the livers of cholestatic mice, and indicated that this was relieved by CBP treatment. Moreover, CBP treatment effectively decreased the liver expression of NF-κB, IKKα, ICAM-1, and IL-1 proteins, and increased IκBα expression in cholestatic mice. The present results suggested that the alleviation of NF-κB-mediated inflammation played an important role in the protective effect of CBP against cholestatic liver injury.

FXR is an important BA-activated nuclear receptor that can modify cholestatic liver injury [42, 43]. FXR activation can also inhibit NF-κΒ pathway-mediated inflammation [44, 45]. Our current results found that CBP treatment induced hepatic FXR expression, promoted expression of its downstream genes (Bsep, Mrp2, and Ugt1a1), reversed disordered BA homeostasis, inhibited NF-κΒ mediated inflammation, and finally reduced cholestatic liver injury. Additionally, luciferase assays conducted in rNtcp-HEK293T cells revealed that CBP could concentration-dependently increase FXR activity. Therefore, we used FXRKO mice to investigate whether the protective effects of CBP against cholestatic liver injury in FXR-dependent. Our findings indicated that CBP could not ameliorate the intrahepatic cholestasis induced by ANIT in FXRKO mice. Taken together, these results suggested that CBP-mediated protection from cholestatic liver injury was FXR-dependent.

In this study, treatment with CBP increased the liver levels of UDCA, TUDCA, CDCA, and TCDCA in cholestatic mice. To investigate the effect of CBP ingredients (TCDCA and TCA) as well as metabolites (TUDCA, UDCA, CDCA and CA) on FXR activity, luciferase assays were conducted using HEK293T cells and rNtcp-HEK293T cells. The results indicated that (except for CDCA), UDCA also activated FXR in HEK293T cells. Additionally, TCDCA, TUDCA, and CBP all activated FXR in rNtcp-HEK293T cells. Quantitation of hepatic cytoplasmic and nuclear BAs revealed that non-conjugated BAs were not detected in rNtcp-HEK293T cells, further confirming the direct effect of TCDCA and TUDCA on FXR. Furthermore, the nuclear concentrations of CDCA, UDCA, TCDCA, and TUDCA were all increased in the livers of cholestatic mice treated with CBP. Taken together, these findings suggested that after treatment with CBP, TCDCA and its derivatives (CDCA, UDCA, and TUDCA) could all activate FXR.

In order to illuminate the effective components of CBP against cholestatic liver injury *in vivo*, the effects of TCDCA and TCA (equivalent to the amounts in 0.2 g/kg CBP) on ANIT-treated mice were investigated. These results showed that TCDCA improved more of the biochemical indicators than TCA. Therefore, TCDCA may be largely responsible for the effects of CBP on ANIT-induced cholestatic liver injury. Additionally, treatment with CBP significantly increased the level of UDCA in the liver and bile of cholestatic mice, but not in the serum. The reasons for this phenomenon may include the following. Firstly, intestinal microbial enzymes have been reported to catalyze the conversion of TCDCA to CDCA, and then to UDCA [46–49], which can be absorbed by hepatocytes. The proportion of TCDCA in CBP was 74% and this treatment may therefore significantly increase the liver level of UDCA. Secondly, CBP treatment markedly increased the liver expression of BA efflux transporters (Bsep and Mrp2). This could enhance biliary BA excretion, which is the major efflux route for hepatic BAs. Therefore, the increased level of UDCA in the liver could be excreted into bile. This would mean that the serum UDCA level would not change significantly, even though CBP slightly increased the BA efflux transporters, Mrp3 and Mrp4. Additionally, increased liver level of CDCA and an increasing trend of FXR expression were found in normal mice after treated with CBP. But CBP had no influence on liver function of normal mice. The reason for this phenomenon may be due to that the bile acid homeostasis is well regulated and controlled in normal mice. However, this is still needed to investigate in the future.

High concentrations of hydrophobic BAs are potentially hepatotoxic and CDCA was reported to significantly reduce cell viability at concentrations above 50 μM, while TCDCA required a higher concentration (250 μM) to produce this effect [50, 51]. The current research found that the liver concentrations of both CDCA and TCDCA (16.6 and 25.8 nmol/g liver) after CBP treatment were far below these cytotoxic concentrations. Our results identified marked increases in the liver levels of UDCA and TUDCA after CBP treatment, which might be derived from the TCDCA following catalysis by intestinal microbial enzymes. However, further research is required to determine the exact biotransformations of BAs following treatment with CBP. Additionally, results from metabolomic studies revealed that CBP and UDCA shared 15 metabolites (Supplementary Table 5 and Supplementary Figure 4). However, there were also some metabolic differences between the two groups, indicating that CBP and UDCA may act via some different mechanisms, which should be explored in the future.

In conclusion, our data highlight the protective effects of CBP in intrahepatic cholestasis and liver injury. The mechanism underlying the effects of CBP involves increasing FXR activation and expression, reversing the disordered homeostasis of BAs, and alleviating liver inflammation. The present results indicated that CBP may provide a natural source for the treatment of intrahepatic cholestasis.

## MATERIALS AND METHODS

### Chemicals and reagents

ANIT, carbamazepine, mycophenolic acid, and reference standards for 18 BAs, including CA, glycocholic acid, TCA, CDCA, glycochenodeoxycholic acid, TCDCA, deoxycholic acid, glycodeoxycholic acid, taurodeoxycholic acid, UDCA, glycoursodeoxycholic acid, TUDCA, LCA, glycolithocholic acid, taurolithocholic acid, hyodeoxycholic acid, glycohyodeoxycholic acid, and taurohyodeoxycholic acid were purchased from Sigma-Aldrich (St. Louis, MO, USA). Chromatography-grade acetonitrile was purchased from Merck (Darmstadt, Germany). Water was purified using a MilliQ water system (Millipore, Bedford, MA, USA). Chromatography-grade acetic acid, formic acid, and methanol were provided by Tedia Company (Fairfield, OH, USA).

### Preparation and composition analysis of CBP

CBP (batch number: SJ141101) was prepared and supplied by Kai Bao Pharmaceutical Co. (Shanghai, China). Briefly, chicken bile was filtered and centrifuged. The supernatant was concentrated and dried to obtain the crude extract, which was dissolved in ethanol. After extraction by ethyl acetate, the aqueous phase was collected, filtered, and dried to produce CBP. The main constituents of CBP were measured by ultra-performance liquid chromatography-quadrupole mass spectrometry (UPLC-MS/MS). The proportions of TCDCA, TCA, CDCA, and CA in CBP were 74, 15, 2.81, and 0.45%, respectively.

### Animals and experimental design

Male wild-type C57BL/6 mice (8 weeks old) weighing 18-20 g were provided by the Animal Center of Shanghai University of Traditional Chinese Medicine (SHUTCM, Shanghai, China). FXRKO mice were obtained from UC Davis Medical Center, and reproduced in a SHUTCM animal feeding room. All mice were kept in a room at 22-24°C, with a light/dark cycle of 12/12 h and 55-60% relative humidity, with free access to standard rodent food and water. All animal experimental procedures were approved by the Committee on the Use of Live Animals for Teaching and Research of the Shanghai University of Traditional Chinese Medicine (Approval Number: 20150401), and all experiments were performed in accordance with the guidelines of this committee.

For animal experiment 1, 40 wild-type mice were randomly assigned to 4 groups: vehicle group, vehicle+ANIT group, ANIT+CBP group (0.2 g/kg, i.g.), and ANIT+UDCA group (115 mg/kg, i.g.). For animal experiment 2, 12 FXRKO mice were divided into 3 groups: vehicle group, ANIT-treated group, and ANIT-treated group that was pretreated with CBP (0.2 g/kg, i.g.). For animal experiment 3, 70 wild-type mice were randomly assigned to 7 groups: vehicle group, vehicle+ANIT group, ANIT+CBP groups that were pretreated with low (0.05 g/kg, i.g.), middle (0.1 g/kg, i.g.), or high (0.2 g/kg, i.g.) doses of CBP, ANIT+TCDCA group (148 mg/kg, i.g.), and ANIT+TCA group (30 mg/kg, i.g.). The drugs were administered to the mice for 14 days, and ANIT (dissolved in corn oil; 50 mg/kg, i.g.) was administered on the 12^th^ day. Forty-eight hours after ANIT treatment, serum and liver tissue samples were obtained, snap-frozen in liquid nitrogen, and stored at -80°C until use.

### Serum biochemistry and hematoxylin-eosin staining

Serum activities of ALT, AST, and ALP, and serum TBA, TBIL, and DBIL were measured using a Hitachi 7080 Chemistry Analyzer (Hitachi Ltd., Tokyo, Japan). The total levels of BAs in the liver, bile, and feces were measured using a commercially available kit (Nanjing Jiancheng Bioengineering Institute, Nanjing, China), in accordance with the manufacturer’s instructions. Liver samples were fixed with 4% paraformaldehyde in phosphate buffer prior to embedding in paraffin. Ten-micrometer sections were stained with hematoxylin and eosin using standard procedures and observed by light microscopy.

### Transmission electron microscopy

Immediately after dissection, liver tissue samples were immersed in 2.5% glutaraldehyde in 0.1 M cacodylate buffer (pH 7.4) overnight, post-fixed in 2.0% osmium tetroxide for 2 h, dehydrated through a series of ethanol solutions, and embedded in Epon. Ultrathin sections (50 nm) of periportal areas, stained with uranyl acetate and lead citrate, were examined under the JEM100CX-α electron microscope (JEOL Ltd., Tokyo, Japan).

### Metabolomics study

#### Sample preparation

An aliquot of 20 μL serum was deproteinized using 100 μL acetonitrile containing internal standards (positive: carbamazepine 20 ng/mL; negative: mycophenolic acid, 320 ng/mL). The sample mixture was vortex-mixed for 3 min and then centrifuged at 16000 rpm for 10 min at 4°C. QC samples were prepared by combining equal aliquots of all serum samples included in the study, which were processed in the same manner as the other serum samples. The QC samples were analyzed before, during, and after each analysis run.

#### Instrumentation and operation conditions

Liquid chromatographic separation was performed using a UPLC system (Dionex, Thermo Fisher Scientific; Sunnyvale, CA, USA). The analytical column was a Waters ACQUITY UPLC BEH C_18_ column (2.1 mm × 100 mm; 1.7 μm) (Waters, Co., Milford, MA, USA). The column and automatic sampler were maintained at 35°C and 4°C, respectively, and the injection volume was 5 μL. The gradient elution was 20 min at a flow rate of 0.3 mL/min, with mobile phases containing water with 0.1% formic acid (A) and acetonitrile with 0.1% formic acid (B). The elution was run on the following schedule: 10% B from 0.1–2 min; a linear increase from 10–40% B over 5 min; a linear increase from 40–80% B over 4 min; a linear increase from 80–90% B over 4 min; 90% B for 0.5 min. At 15.5 min, B was adjusted to 10% and equilibrated for 4.5 min. The UPLC system was connected to an LTQ-orbitrap elite mass spectrometry system (Thermo Fisher Scientific; Bremen, Germany) via a heated electrospray ionization (ESI) source in both positive and negative ionization mode. The mass spectrometer parameters were as follows: ion spray voltage, 3.8 kV(+) and 3.2 kV(-); capillary and heater temperature, both 350°C; sheath and auxiliary gas flow rate, 45 and 15 psi; and S-Lens RF level, 60%.

#### Data processing and standardization of metabolites, identification of potential biomarkers, and metabolic pathway analysis

The data preprocessing and analysis, identification of potential biomarkers, and pathway analysis were performed as previously reported [52].

### Quantitation of BAs in serum, liver, and bile

UPLC-MS/MS was used to detect 18 BAs in mouse serum, liver, and bile. The sample preparation and UPLC-MS/MS analyses were performed as described previously [53, 54], with slight modification. BA calibration curves were shown in Supplementary Table 7. The cytoplasmic and nuclear levels of CDCA, UDCA, TCDCA, TUDCA, CA, and TCA in HEK293T cells and liver tissues were also determined using UPLC-MS/MS.

### Real-time PCR analysis

Total RNA was isolated from mice livers using TRIzol^®^ and reverse transcribed by a TAKARA cDNA synthesis kit (TAKARA). The resulting cDNA was subjected to quantitative PCR analysis using SYBR^®^ Green and ABI-StepOnePlus Sequence Detection System (Applied Biosystems, CA, USA). Primer sequences are listed in Supplementary Table 8. The relative mRNA expression was calculated using the 2^-ΔΔCt^ method. The expression of glyceraldehyde 3-phosphate dehydrogenase mRNA was used as the endogenous reference control.

### Western blot analysis

Total proteins was extracted from liver tissues, resolved by sodium dodecyl sulfate-polyacrylamide gel electrophoresis, and transferred to polyvinylidene fluoride membranes, which were subsequently probed with primary antibodies raised against Bsep, Mrp2, Mrp3, FXR, ICAM-1 (Santa Cruz, CA, USA), CAR (ABCAM, MA, USA), NF-κB, IκBα, and IKKα (Cell Signaling Technology, MA, USA). The membranes were then incubated with the appropriate horseradish peroxidase-conjugated secondary antibodies. The protein bands were detected using the FluorChem E image detection system (ProteinSimple, Santa Clara, CA, USA). Densitometric analysis was performed and the results were expressed as the integrated optical density, relative to β-actin.

### Immunohistochemistry

Mice livers were fixed in 10% neutral buffered paraformaldehyde at 4°C for 48 h prior to embedding in paraffin, sectioning, and staining with an anti-Mrp2 antibody (1:500; Santa-Cruz). The sections were finally mounted using DPX Mountant (Sigma, MO, USA) for histological analysis.

### Immunofluorescence

Tissue sections were incubated with a primary antibody (rabbit anti-FXR, 1:200; Santa Cruz) and an Alexa 555-conjugated secondary antibody (1:200; Molecular Probes) to produce an immunofluorescent signal. Sections were counterstained with hematoxylin or 4',6-diamidino-2-phenylindole (eBioscience; 00-4959). Positive staining was quantified manually or using Image-Pro Plus 6 Windows Software (Media Cybernetics, USA).

### Cell culture

HEK293T cells were purchased from the Cell Bank of the Shanghai Institutes of Biological Sciences and cultured in normal Dulbecco’s modified Eagle’s medium containing 10% fetal bovine serum at 37°C in an atmosphere containing 5% CO_2_.

### Transient transfection and dual luciferase reporter assay

HEK293T cells were plated onto 48-well plates at a density of 5 × 10^5^ cells/mL for 24 h prior to transfection, and cultured overnight. Cells at 50-70% confluence were transiently transfected in OPTI-MEM reduced-serum medium (Invitrogen) with a mixture of pFXRE-tk-Luc, pCMV-FXR, and pSG5-retinoid X receptor α expression plasmids (1 μg of each), and the pRL-SV40 Renilla plasmid (0.1 μg; Promega) as an internal control for transfection efficiency. All transfections were performed using the Fugene HD transfection reagent (Roche). At 24 h post-transfection, cells were exposed to the indicated treatments in fresh Dulbecco’s modified Eagle’s medium containing 10% fetal bovine serum for an additional 24 h. After treatment, the cells were lysed and luciferase activity was determined using the Dual-Luciferase^®^ Reporter Assay System (Promega, USA), according to the manufacturer’s protocol. Luminescence was measured by the FB12 luminometer (Berthold, Germany). The firefly luciferase activity was normalized to the Renilla luciferase activity for each well. All experiments were conducted in triplicate and were repeated at least twice.

### rNtcp-transfected HEK293T cells and BA uptake

rNtcp-expressing HEK293T cells were obtained by transient transfection with plasmids, as described previously [55]. Luciferase activity was repeatedly determined to compare transfected and non-transfected cells. The uptake of BAs or CBP by rNtcp-HEK293T cells and the intracellular distribution of taurine-conjugated BAs were determined. Twenty-four hours after transfection, the cells were incubated in medium at 37°C for 10 min prior to adding the indicated concentrations of BAs or CBP and incubating for 15 min. The cytoplasmic and nuclear fractions of these cells, and those of the liver tissue obtained from the *in vivo* study, were separated using the NE-PER nuclear and cytoplasmic extraction reagent (Thermo Scientific, USA). The BAs were then determined using UPLC-MS/MS, as described above.

### Statistical analysis

Data are expressed as the mean ± the standard deviation (SD). The statistical differences between the study groups were determined by one-way ANOVA analysis. For all comparisons, p < 0.05 was considered to represent a statistically significant difference.

## SUPPLEMENTARY MATERIALS FIGURES AND TABLES


