# Supplementary material for: Chicken bile powder protects against α-naphthylisothiocyanate-induced cholestatic liver injury in mice
Source: Oncotarget. 2017 Sep 27;8(57):97137–52. doi: 10.18632/oncotarget.21385 (PMC5722551; doi:10.18632/oncotarget.21385)
Supplement: Supplementary file 1 [file oncotarget-08-97137-s001.pdf]

Chicken bile powder protects against α-naphthylisothiocyanate-induced cholestatic liver injury in mice

SUPPLEMENTARY MATERIALS

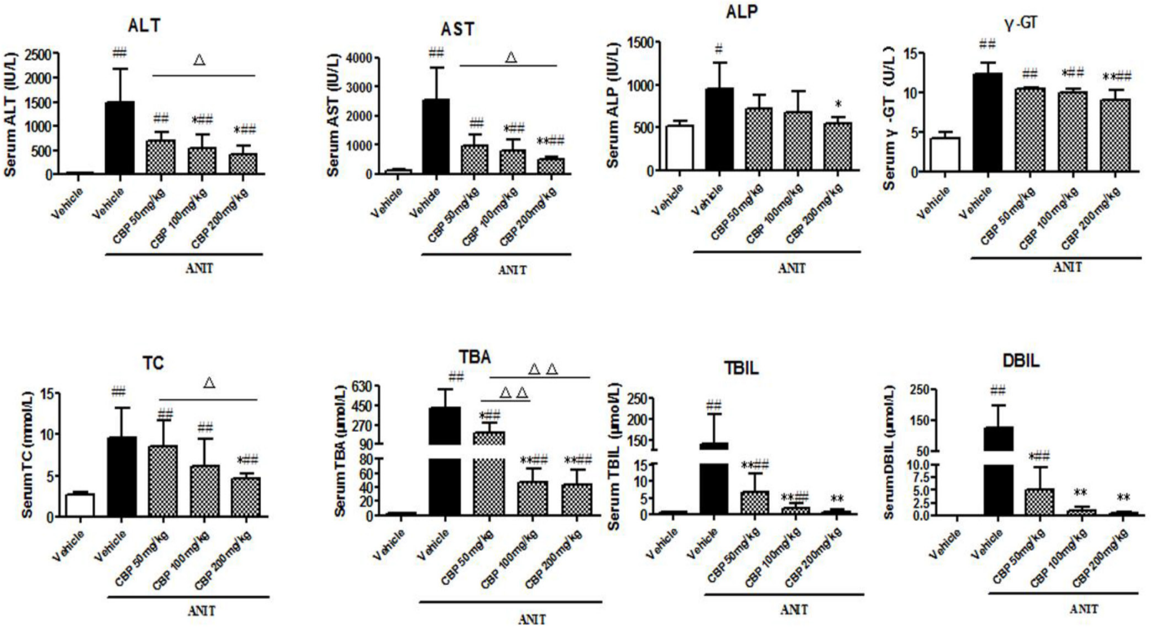

Supplementary Figure 1: Effects of different dose of CBP on ANIT-induced intrahepatic cholestasis in mice. Data are expressed as the mean ± S.D., n = 10; $^{*}p < 0.05$, $^{**}p < 0.01$ for the comparison with the vehicle + ANIT group. $^{\#}p < 0.05$, $^{\#\#}p < 0.01$ for the comparison with the vehicle group; $^{\Delta}p < 0.05$, $^{\Delta\Delta}p < 0.01$ for the comparison with the ANIT+ CBP 50 mg/kg group.

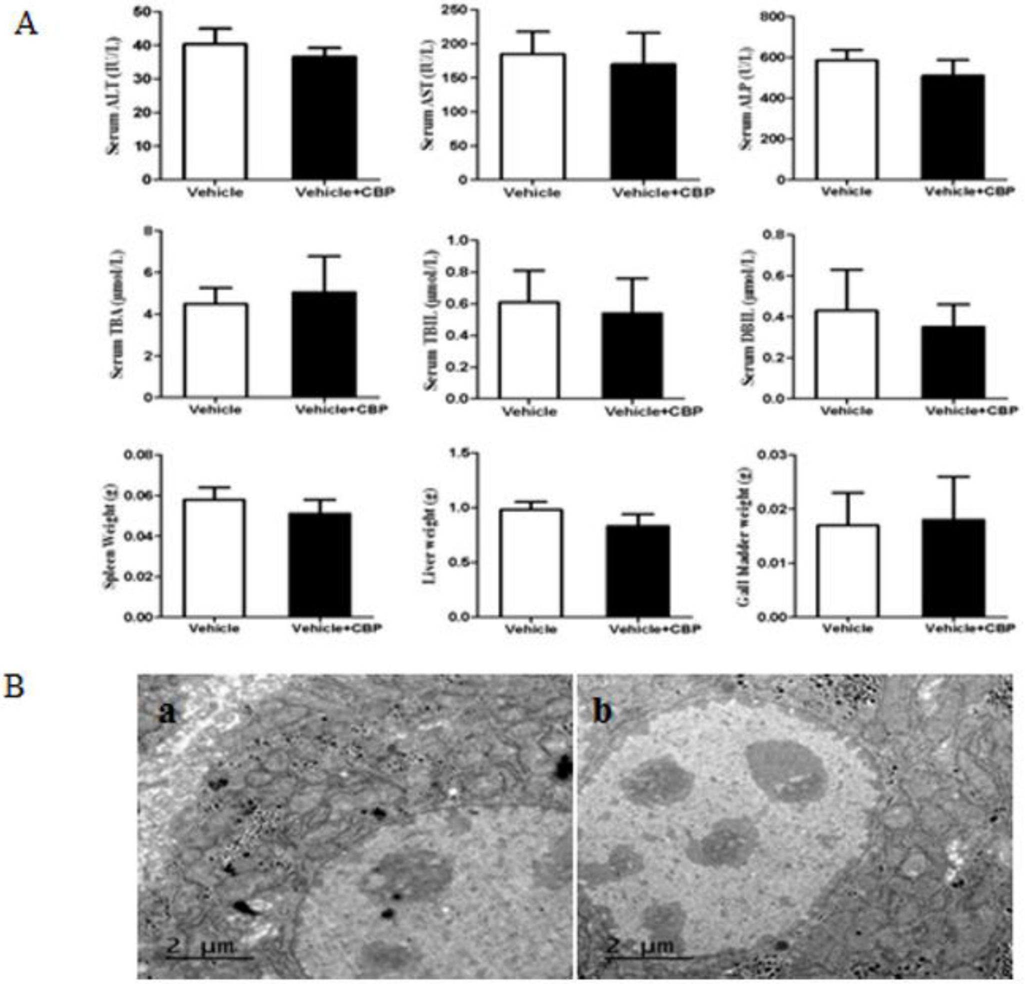

Supplementary Figure 2: The effect of CBP on liver function and ultrastructure in normal mice. **(A)** Serum biochemical parameters. **(B)** Liver microstructure was observed by electron microscopy (scale bar 2 μm, original magnification × 6000), a: normal control, b: normal mice treated with CBP. Data are expressed as the mean ± S.D., n = 10 for serum biochemical parameters and n = 3 for electron microscopy.

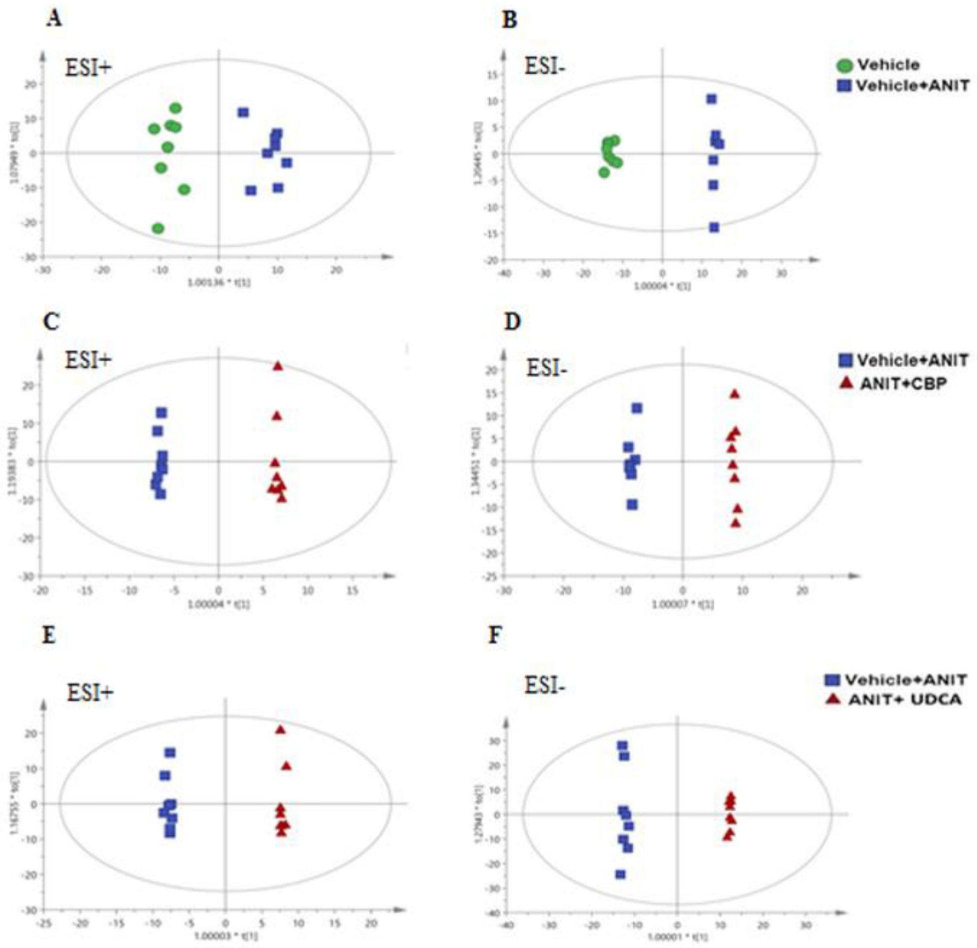

Supplementary Figure 3: The OPLS-DA score plots between vehicle and vehicle+ANIT group, vehicle+ANIT and ANIT+CBP group, and vehicle+ANIT and ANIT+UDCA group. **(A)** ESI+ mode between Vehicle and Vehicle+ANIT groups, $R^2X = 0.878$, $R^2Y = 0.986$, $Q^2= 0.946$; **(B)** ESI- mode between Vehicle and Vehicle+ANIT groups, $R^2X = 0.819$, $R^2Y = 0.996$, $Q^2 = 0.956$. **(C)** ESI+ mode between Vehicle+ANIT and ANIT+ CBP groups, $R^2X = 0.883$, $R^2Y = 0.998$, $Q^2 = 0.889$; **(D)** ESI- mode between Vehicle+ANIT and ANIT+ CBP groups, $R^2X = 0.830$, $R^2Y = 0.997$, $Q^2 = 0.786$; **(E)** ESI+ mode between Vehicle+ANIT and ANIT+ UDCA groups, $R^2X = 0.883$, $R^2Y = 0.998$, $Q^2 = 0.878$; **(F)** ESI- mode between Vehicle+ANIT and ANIT+ UDCA groups, $R^2X = 0.945$, $R^2Y = 0.998$, $Q^2 = 0.967$.

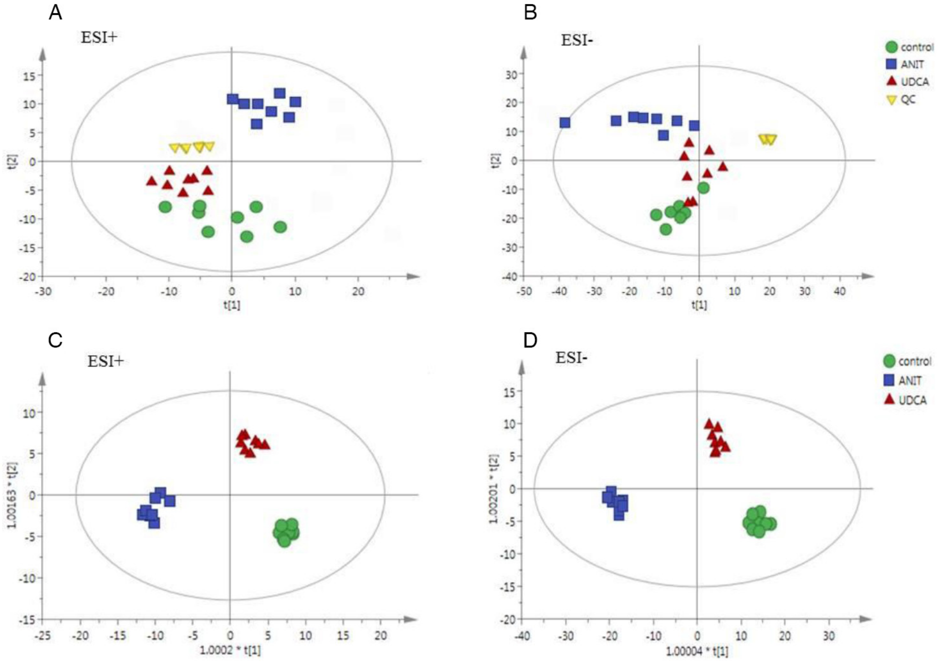

Supplementary Figure 4: The PCA and OPLS-DA score plots of UDCA in mice with intrahepatic cholestasis. PCA score plot in the indicated study groups in **(A)** ESI+ mode, $R^2X = 0.955$, $Q^2 = 0.839$; **(B)** ESI- mode, $R^2X = 0.954$, $Q^2 = 0.897$. OPLS-DA score plot in the indicated study groups in ESI+ mode **(C)** $R^2X = 0.894$, $R^2Y = 0.974$, $Q^2 = 0.900$; and in ESI- mode **(D)** $R^2X = 0.947$, $R^2Y = 0.985$, $Q^2 = 0.802$.

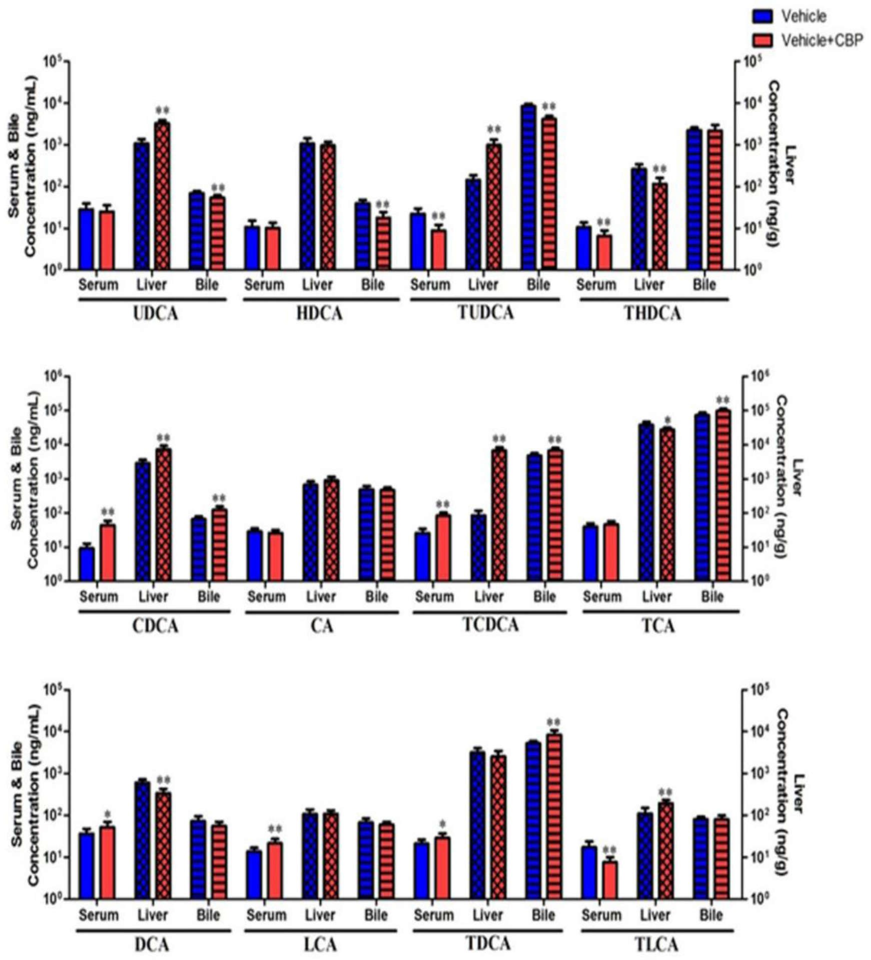

Supplementary Figure 5: Effects of CBP on BA levels in normal mice. The concentration of BA components in serum, liver, and bile. Data are expressed as the mean ± S.D., n = 10; $^{*}p < 0.05$, $^{**}p < 0.01$ for the comparison with the vehicle group.

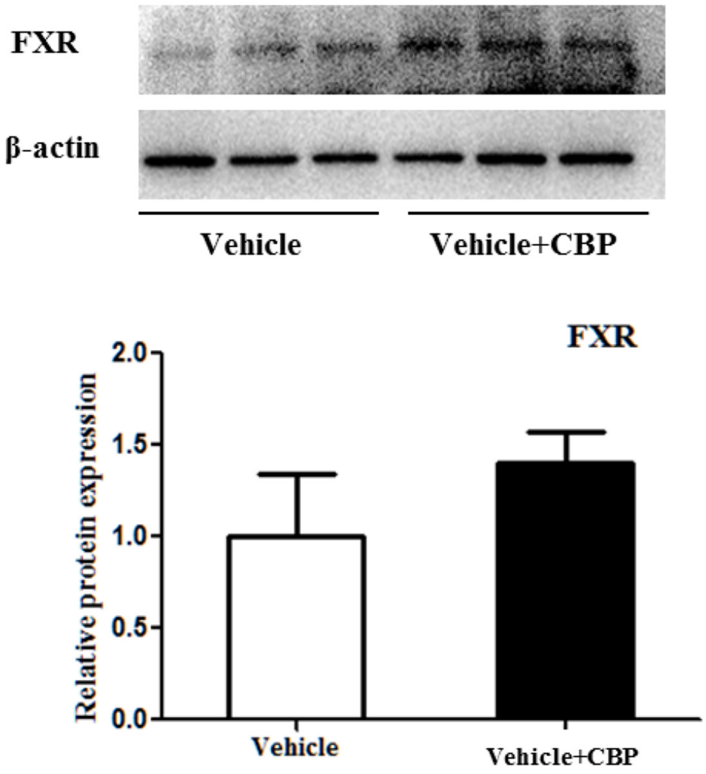

Supplementary Figure 6: The effect of CBP on liver FXR expression in normal mice. Data are expressed as the mean ± S.D., n =3.

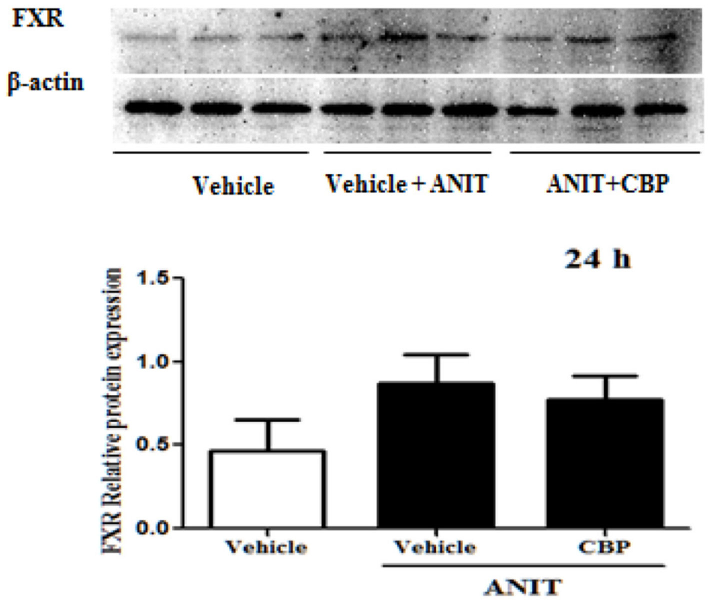

Supplementary Figure 7: The effect of CBP on liver FXR expression at 24h in cholestatic mice. Data are expressed as the mean ± S.D., n =3.

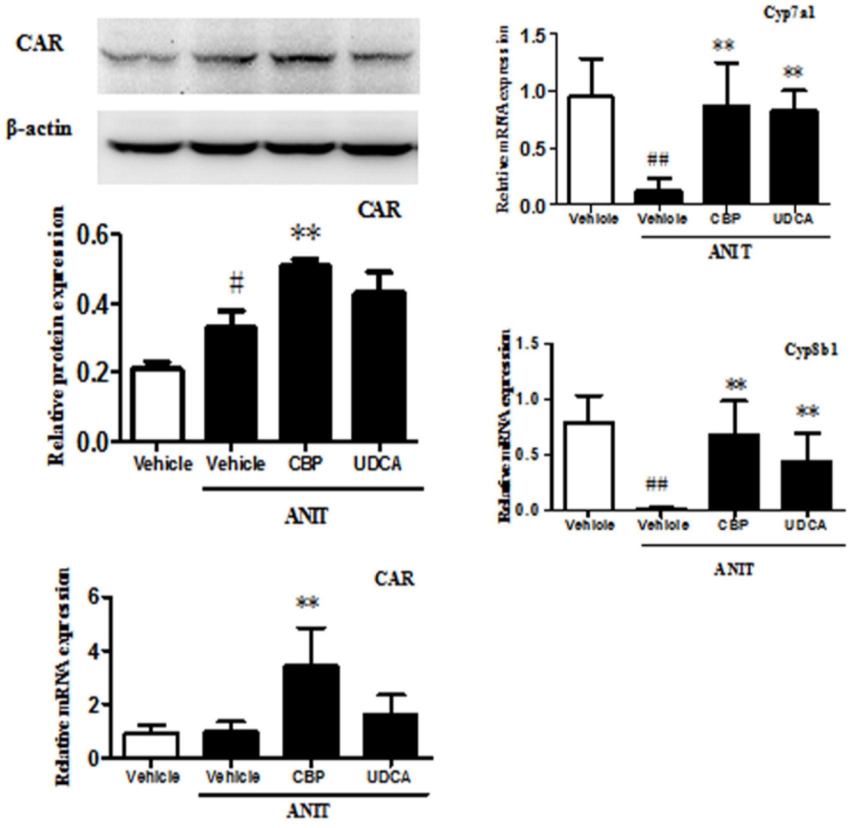

Supplementary Figure 8: Effects of CBP on nuclear receptor CAR and BA metabolic enzyme expression in mice with intrahepatic cholestasis. Data are expressed as the mean ± S.D., n = 5; $^{\#}p < 0.05$, $^{\#\#}p < 0.01$ for the comparison with the vehicle group; $^{*}p < 0.05$, $^{**}p < 0.01$ for the comparison with the vehicle + ANIT group.

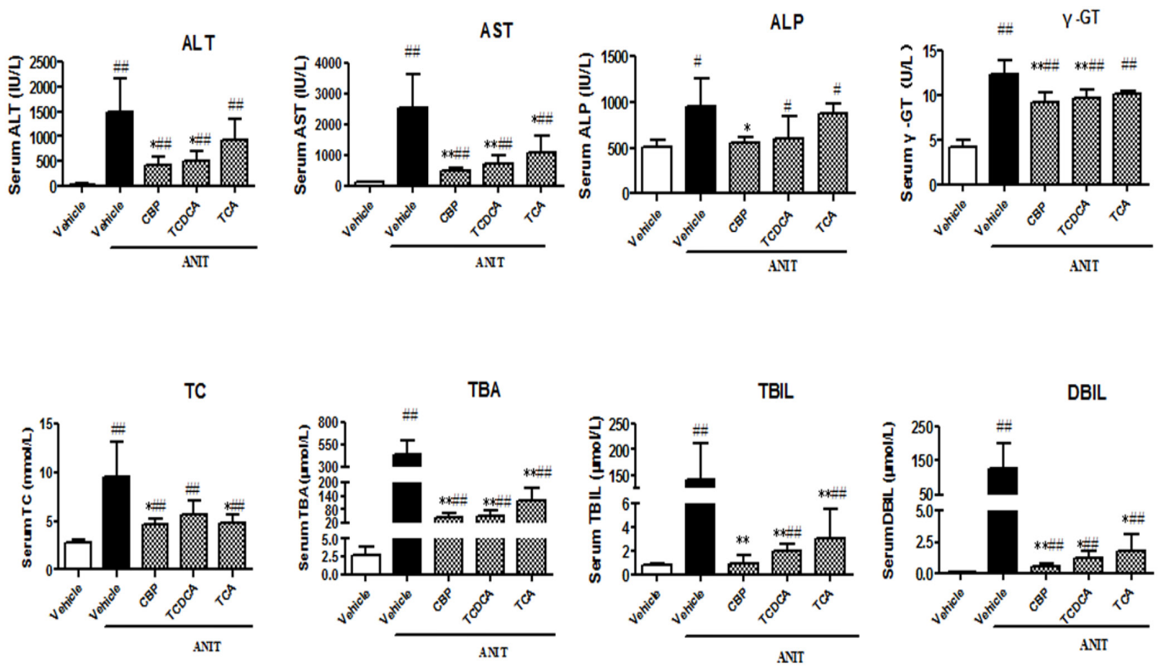

Supplementary Figure 9: The effect of CBP main ingredients on liver function in ANIT-induced cholestatic mice. Data are expressed as the mean ± S.D., n = 10 for serum biochemical parameters; $^{\#}p < 0.05$, $^{\#\#}p < 0.01$ for the comparison with the vehicle group; $^{*}p < 0.05$, $^{**}p < 0.01$ for the comparison with the vehicle + ANIT group.

Supplementary Table 1: The repeatability and precision (RSD) of QC samples in the UPLC-LTQ-Orbitrap analysis

Mode	Biomarkers	Stability(0h,24h)(n=8)			Inner-precision			Intra-precision		
		Mean	SD	RSD	Mean	SD	RSD	Mean	SD	RSD
ESI+	Pyroglutamic acid	0.2179	0.0144	0.0660	0.2365	0.0122	0.0516	0.2291	0.0120	0.0523
	Methionine	0.7044	0.0424	0.0602	0.7470	0.0160	0.0214	0.7214	0.0326	0.0452
	Linoleic acid	0.7931	0.0774	0.0975	0.8240	0.0497	0.0603	0.8005	0.0591	0.0738
	Taurocholic acid	0.7402	0.0760	0.1027	0.7736	0.0486	0.0628	0.7528	0.0643	0.0854
	LysoPC(18:2)	11.6889	0.9047	0.0774	11.3981	0.4571	0.0401	11.6812	0.7618	0.0652
	LysoPC(20:1)	0.6499	0.0614	0.0945	0.7163	0.0293	0.0409	0.6824	0.0480	0.0704
ESI-	Prostaglandin F2α	0.0360	0.0032	0.0888	0.0387	0.0028	0.0714	0.0377	0.0027	0.0713
	Cholic acid	0.1740	0.0166	0.0955	0.1928	0.0055	0.0286	0.1815	0.0139	0.0764
	Bilirubin	0.0048	0.0006	0.1267	0.0056	0.0003	0.0513	0.0052	0.0006	0.1156

Supplementary Table 2: The endogenous serum metabolites identified in normal control and ANIT-induced model control mice

VIP	Time (min)	Molecular ion	Compound MW	MZ	Formula	Metabolites
1.2371	0.87	$[M+H]^+$	131.0688	132.0761	$C_4H_9N_3O_2$	Creatine
1.9915	0.95	$[M+H]^+$	149.0503	150.0575	$C_5H_{11}NO_2S$	Methionine
1.4261	0.99	$[M+Na]^+$	204.0889	227.0777	$C_{11}H_{12}N_2O_2$	Tryptophan
1.3400	3.49	$[M+H]^+$	173.1044	174.1117	$C_8H_{15}NO_3$	N-caproylglycine
3.9315	3.77	$[M+H]^+$	515.2887	516.2960	$C_{26}H_{45}NO_7S$	Taurocholic acid
2.6512	3.91	$[M-H]^-$	513.2745	512.2678	$C_{26}H_{43}NO_7S$	Sulfolithocholylglycine
2.3625	4.40	$[M+H]^+$	362.2077	363.2149	$C_{21}H_{30}O_5$	Cortisol
1.5510	4.41	$[M-H]^-$	362.2084	361.2015	$C_{21}H_{30}O_5$	Cortisol
1.9255	4.58	$[M-H]^-$	408.2865	407.2794	$C_{24}H_{40}O_5$	Cholic acid
3.1122	4.93	$[M+H]^+$	584.2606	585.2679	$C_{33}H_{36}N_4O_6$	Bilirubin
3.7841	5.04	$[M+H]^+$	499.2948	500.3046	$C_{26}H_{45}NO_6S$	Taurochenodeoxycholic acid
1.2292	5.46	$[M-H]^-$	354.2397	353.2335	$C_{20}H_{34}O_5$	Prostaglandin F2α
1.0568	6.03	$[M-H]^-$	188.1411	187.1339	$C_{10}H_{20}O_3$	10-Hydroxydecanoic acid
1.4079	6.92	$[M+H]^+$	379.2470	380.2542	$C_{18}H_{38}NO_5P$	Sphingosine 1-phosphate
1.0619	6.92	$[M-H]^-$	379.2477	378.2406	$C_{18}H_{38}NO_5P$	Sphingosine 1-phosphate
2.3307	7.10	$[M+H]^+$	541.3142	542.3215	$C_{28}H_{48}NO_7P$	LysoPC(20:5)
1.7150	7.41	$[M+H]^+$	481.3145	482.3218	$C_{23}H_{48}NO_7P$	LysoPC(15:0)
1.4450	7.54	$[M+H]^+$	519.3282	520.3354	$C_{26}H_{50}NO_7P$	LysoPC(18:2)
1.5763	7.67	$[M+H]^+$	567.3278	568.3351	$C_{30}H_{50}NO_7P$	LysoPC(22:6)
1.8473	7.97	$[M+H]^+$	569.3453	570.3525	$C_{30}H_{52}NO_7P$	LysoPC(22:5)
1.5650	8.40	$[M-H]^-$	320.2344	319.2276	$C_{20}H_{32}O_3$	8,9-Epoxyeicosatrienoic acid
1.5343	8.61	$[M+H]^+$	399.3314	400.3387	$C_{23}H_{45}NO_4$	L-Palmitoylcarnitine
1.5513	8.71	$[M+H]^+$	509.3462	510.3535	$C_{25}H_{52}NO_7P$	LysoPC(17:0)
1.8644	9.95	$[M+H]^+$	549.3771	550.3844	$C_{28}H_{56}NO_7P$	LysoPC(20:1)
1.0999	11.16	$[M+H]^+$	330.2546	331.2619	$C_{22}H_{34}O_2$	Docosapentaenoic acid
2.2003	11.60	$[M+H]^+$	551.3929	552.4002	$C_{28}H_{58}NO_7P$	LysoPC(20:0)

Supplementary Table 3: The endogenous serum metabolites identified in ANIT-induced mice treated with or without CBP

VIP	Time (min)	Molecular ion	Compound MW	MZ	Formula	Metabolites
1.8850	0.76	$[M+H]^+$	132.0893	133.0965	$C_5H_{12}N_2O_2$	Ornithine
1.6319	0.78	$[M+H]^+$	145.1572	146.1645	$C_7H_{19}N_3$	Spermidine
1.1169	0.84	$[M-H]^-$	146.0693	145.0621	$C_5H_{10}N_2O_3$	Glutamine
1.2371	0.87	$[M+H]^+$	131.0688	132.0761	$C_4H_9N_3O_2$	Creatine
1.9915	0.95	$[M+H]^+$	149.0503	150.0575	$C_5H_{11}NO_2S$	Methionine
1.0753	0.96	$[M+H]^+$	164.0465	165.0538	$C_9H_8O_3$	Phenylpyruvic acid
1.8961	0.97	$[M+H]^+$	129.0422	130.0494	$C_5H_7NO_3$	Pyroglutamic acid
1.0874	0.97	$[M+OH]^-$	129.0427	146.0457	$C_5H_7NO_3$	Pyroglutamic acid
1.9199	1.53	$[M-H]^-$	213.0092	212.0020	$C_8H_7NO_4S$	3-Indoxylsulfate
2.1714	2.91	$[M-H]^-$	179.0579	178.0510	$C_9H_9NO_3$	Hippuric acid
1.4057	3.17	$[M-H]^-$	193.0735	192.0664	$C_{10}H_{11}NO_3$	2-Methylhippuric acid
3.9315	3.77	$[M+H]^+$	515.2887	516.2960	$C_{26}H_{45}NO_7S$	Taurocholic acid
2.6512	3.91	$[M-H]^-$	513.2745	512.2678	$C_{26}H_{43}NO_7S$	Sulfolithocholylglycine
2.3625	4.40	$[M+H]^+$	362.2077	363.2149	$C_{21}H_{30}O_5$	Cortisol
1.5510	4.41	$[M-H]^-$	362.2084	361.2015	$C_{21}H_{30}O_5$	Cortisol
1.9255	4.58	$[M-H]^-$	408.2865	407.2794	$C_{24}H_{40}O_5$	Cholic acid
3.1122	4.93	$[M+H]^+$	584.2606	585.2679	$C_{33}H_{36}N_4O_6$	Bilirubin
3.7841	5.04	$[M+H]^+$	499.2948	500.3046	$C_{26}H_{45}NO_6S$	Taurochenodeoxycholic acid
1.2292	5.46	$[M-H]^-$	354.2397	353.2335	$C_{20}H_{34}O_5$	Prostaglandin F2α
1.7293	5.80	$[M-H]^-$	392.2911	391.2845	$C_{24}H_{40}O_4$	Chenodeoxycholic acid
1.4079	6.92	$[M+H]^+$	379.2470	380.2542	$C_{18}H_{38}NO_5P$	Sphingosine 1-phosphate
1.0619	6.92	$[M-H]^-$	379.2477	378.2406	$C_{18}H_{38}NO_5P$	Sphingosine 1-phosphate
1.1369	6.95	$[M-H]^-$	216.1721	215.1650	$C_{12}H_{24}O_3$	b-Hydroxylauric acid
2.3307	7.10	$[M+H]^+$	541.3142	542.3215	$C_{28}H_{48}NO_7P$	LysoPC(20:5)
1.5587	7.13	$[M+H]^+$	493.3148	494.3221	$C_{24}H_{48}NO_7P$	LysoPC(16:1)
1.5150	7.43	$[M+H]^+$	299.2809	300.2882	$C_{18}H_{37}NO_2$	N-(2-Hydroxyethyl)hexadecanamide
1.4450	7.54	$[M+H]^+$	519.3282	520.3354	$C_{26}H_{50}NO_7P$	LysoPC(18:2)
1.5763	7.67	$[M+H]^+$	567.3278	568.3351	$C_{30}H_{50}NO_7P$	LysoPC(22:6)
1.8473	7.97	$[M+H]^+$	569.3453	570.3525	$C_{30}H_{52}NO_7P$	LysoPC(22:5)
1.5650	8.40	$[M-H]^-$	320.2344	319.2276	$C_{20}H_{32}O_3$	8,9-Epoxyeicosatrienoic acid
1.5343	8.61	$[M+H]^+$	399.3314	400.3387	$C_{23}H_{45}NO_4$	L-Palmitoylcarnitine
1.4141	8.68	$[M-H]^-$	322.2498	321.2428	$C_{20}H_{34}O_3$	15S-Hydroxyeicosatrienoic acid
2.1205	8.89	$[M+H]^+$	547.3613	548.3686	$C_{28}H_{54}NO_7P$	LysoPC(20:2)
1.5752	10.60	$[M+H]^+$	328.2390	329.2463	$C_{22}H_{32}O_2$	Docosahexaenoic acid
1.2507	11.26	$[M+H]^+$	280.2392	281.2465	$C_{18}H_{32}O_2$	Linoleic acid
1.5836	11.77	$[M+H]^+$	306.2545	307.2617	$C_{20}H_{34}O_2$	8Z,11Z,14Z-Eicosatrienoic acid

Supplementary Table 4: The major pathways identified in the metabolomics study as influenced by CBP

Pathway name	Total	Hits	p	-log(p)	Impact
Primary bile acid biosynthesis	46	3	0.0073	4.9209	0.0595
Arachidonic acid metabolism	36	2	0.0411	3.1926	0.0208
Taurine and hypotaurine metabolism	8	1	0.0713	2.6415	0.0000
Sphingolipid metabolism	21	1	0.1771	1.7310	0.0301
Porphyrin and chlorophyll metabolism	27	1	0.2221	1.5046	0.0415
Cysteine and methionine metabolism	27	1	0.2221	1.5046	0.0869
Glycerophospholipid metabolism	30	1	0.2438	1.4116	0.0444
Glycine, serine and threonine metabolism	31	1	0.2508	1.3830	0.0000
Fatty acid metabolism	39	1	0.3054	1.1862	0.0000
Arginine and proline metabolism	44	1	0.3376	1.0860	0.0120
Aminoacyl-tRNA biosynthesis	69	1	0.4789	0.7363	0.0000
Steroid hormone biosynthesis	72	1	0.4938	0.7056	0.0539

Supplementary Table 5: The endogenous serum metabolites identified in ANIT-induced mice treated with or without UDCA

VIP	Time (min)	Molecular ion	Compound MW	MZ	Formula	Metabolites
2.1442	0.76	$[M+H]^+$	132.0893	133.0965	$C_5H_{12}O_2N_2$	Ornithine
1.2360	0.87	$[M+H]^+$	131.0688	132.0761	$C_4H_9N_3O_2$	Creatine*
1.5373	0.96	$[M+H]^+$	129.0422	130.0494	$C_5H_7O_3N$	Pyroglutamic acid
2.0705	1.06	$[M-H]^-$	88.0161	87.0089	$C_3H_4O_3$	Pyruvic acid
3.9838	3.77	$[M+H]^+$	515.2887	516.2960	$C_{26}H_{45}NO_7S$	Taurocholic acid*
2.4850	4.40	$[M+H]^+$	362.2077	363.2149	$C_{21}H_{30}O_5$	Cortisol*
3.1559	4.55	$[M-H]^-$	499.2954	498.2891	$C_{26}H_{45}NO_6S$	Tauroursodeoxycholic acid
2.3135	4.93	$[M+H]^+$	584.2606	585.2679	$C_{33}H_{36}N_4O_6$	Bilirubin*
2.3552	5.46	$[M-H]^-$	354.2399	353.2329	$C_{20}H_{34}O_5$	Prostaglandin F2α*
2.3257	5.80	$[M-H]^-$	392.2918	391.2845	$C_{24}H_{40}O_4$	Ursodeoxycholic acid
1.4392	6.92	$[M+H]^+$	379.2470	380.2542	$C_{18}H_{38}NO_5P$	Sphingosine 1-phosphate*
2.3409	7.10	$[M+H]^+$	541.3142	542.3215	$C_{28}H_{48}NO_7P$	LysoPC(20:5)*
2.0397	7.21	$[M+H]^+$	517.3137	518.3210	$C_{26}H_{48}NO_7P$	LysoPC(18:3)
1.3837	7.54	$[M+H]^+$	519.3276	520.3348	$C_{26}H_{50}NO_7P$	LysoPC(18:2)*
1.6550	8.05	$[M-H]^-$	256.2400	255.2329	$C_{16}H_{32}O_2$	Palmitic acid
1.7084	8.61	$[M+H]^+$	399.3314	400.3387	$C_{23}H_{45}NO_4$	L-Palmitoylcarnitine*
1.6136	10.60	$[M+H]^+$	328.2390	329.2463	$C_{22}H_{32}O_2$	Docosahexaenoic acid
1.4464	11.16	$[M+H]^+$	330.2545	331.2617	$C_{22}H_{34}O_2$	Docosapentaenoic acid
2.2000	11.60	$[M+H]^+$	551.3929	552.4002	$C_{28}H_{58}NO_7P$	LysoPC(20:0)

*These metabolites are same as the results of CBP.

Supplementary Table 6: Effects of CBP on nuclear BAs in rNtcp-HEK293T cells (ng/mL)

	TUDCA		TCDCA		TCA		CBP	
	20 μM	40μM	20 μM	40μM	20 μM	40μM	20 μM	40μM
TUDCA	1260.17±96.95	1300.00±43.59	/	/	/	1.82±0.47	/	/
UDCA	/	/	/	/	/	/	/	/
TCDCA	120.20±8.45	90.00±3.10	965.00±26.85	1151.33±97.9	79.20±4.69	73.83±10.06	720.33±97.27	860.40±46.41
CDCA	/	/	/	/	/	/	/	/
TCA	18.93±5.74	19.97±1.48	11.11±1.13	10.75±0.31	1330.00±72.11	1376.67±171.56	56.43±14.35	41.93±3.61
CA	/	/	/	/	/	/	/	/

Data are expressed as mean ± S.D., n=3./: BA component was below the lower limit of quantification.

Supplementary Table 7: Calibration curves for BA quantitation in serum, liver, and bile

Bile acids	serum			liver			bile		
	Intercept	Slope	R^2	Intercept	Slope	R^2	Intercept	Slope	R^2
TUDCA	0.000171	0.000107	0.9971	0.00547	0.000492	0.9964	0.00000865	0.000126	0.9973
TCDCA	0.00111	0.0006	0.9950	0.00307	0.000511	0.9969	-0.000196	0.00038	0.9978
THDCA	0.000604	0.000466	0.9988	0.00187	0.000505	0.9970	-0.00000567	0.00034	0.9970
TDCA	-0.000257	0.000356	0.9953	0.000916	0.000759	0.9947	-0.0005	0.00031	0.9921
TCA	0.0000958	0.000107	0.9965	0.0123	0.000402	0.9958	0.000474	0.0000943	0.9962
TLCA	0.00011	0.000509	0.9949	0.00102	0.000682	0.9952	0.0000583	0.000293	0.9958
UDCA	0.00411	0.00187	0.9962	0.00542	0.00193	0.9953	0.000186	0.00111	0.9986
CDCA	0.00773	0.00106	0.9977	0.0275	0.00174	0.9948	0.00253	0.000939	0.9982
HDCA	0.00744	0.00188	0.9972	0.000548	0.00211	0.9945	0.000831	0.00145	0.9990
DCA	0.00574	0.00492	0.9950	0.0259	0.00547	0.9977	0.00517	0.00323	0.9969
CA	0.00225	0.00112	0.9960	0.00313	0.00433	0.9929	0.000639	0.00106	0.9979
LCA	0.000182	0.00135	0.9976	0.103	0.00146	0.9960	0.0193	0.00104	0.9989
GUDCA	0.000751	0.000451	0.9977	0.0000411	0.000785	0.9953	0.000102	0.000407	0.9985
GHDCA	0.00122	0.000542	0.9974	0.00039	0.000696	0.9905	-0.000583	0.000545	0.9972
GCDCA	0.00026	0.0000895	0.9959	0.0000892	0.000452	0.9956	-0.0000723	0.000133	0.9992
GDCA	-0.000142	0.000692	0.9950	-0.00000284	0.000656	0.9949	-0.000352	0.000547	0.9966
GCA	0.000174	0.00017	0.9946	0.0000951	0.000477	0.9960	-0.0000147	0.000152	0.9959
GLCA	-0.0000692	0.000152	0.9924	-0.0000497	0.000395	0.9940	-0.000121	0.000208	0.9977

Supplementary Table 8: Sequences of the Real-time PCR primers used in this study

Gene	Specise	Gene bank	Primer sequence (5'-3')
Gapdh	Mouse	NM_008084	Forward primer AGGTCGGTGTGAACGGATTTG
			Reverse primer GGGGTCGTTGATGGCAACA
Bsep	Mouse	NM_021022	Forward primer TCTGACTCAGTGATTCTTCGCA
			Reverse primer CCCATAAACATCAGCCAGTTGT
Mrp2	Mouse	NM_013806	Forward primer GTGTGGATTCCCTTGGGCTTT
			Reverse primer CACAACGAACACCTGCTTGG
Mrp3	Mouse	NM_029600	Forward primer CTGGGTCCCCTGCATCTAC
			Reverse primer GCCGTCTTGAGCCTGGATAAC
Mrp4	Mouse	NM_001033336	Forward primer CATCGCGGTAACCGTCCTC
			Reverse primer CCGCAGTTTTACTCCGCAG
Cyp7a1	Mouse	NM_007824	Forward primer GAACCTCCTTTGGACAACGGG
			Reverse primer GGAGTTTGTGATGAAGTGGACAT
Cyp2b10	Mouse	NM_009999	Forward primer TGCTGTCGTTGAGCCAACC
			Reverse primer CCACTAAACATTGGGCTTCCT
Ugt1a1	Mouse	NM_201645	Forward primer GCTTCTTCCGTACCTTCTGTTG
			Reverse primer GCTGCTGAATAACTCCAAGCAT
FXR	Mouse	NM_001163504	Forward primer GCTTGATGTGCTACAAAAGCTG
			Reverse primer CGTGGTGATGGTTGAATGTCC
ICAM-1	Mouse	NM_010493	Forward primer GCCTTGGTAGAGGTGACTGAG
			Reverse primer GACCGGAGCTGAAAAGTTGTA
IL-1β	Mouse	NM_008361	Forward primer ACTCCTTAGTCCTCGGCCA
			Reverse primer CCATCAGAGGCAAGGAGGAA
CYP8b1	Mouse	NM_010012	Forward primer GAATCTAACCAGGCCATGCT
			Reverse primer AGGAGCTGGCACCTAGACT
CAR	Mouse	NM_001243062	Forward primer TTCAAGCCTCCGGCCTATCT
			Reverse primer TGATCTGTTGCACCATAAACGTG
